# Supplementary material for: NeXus: An Automated Platform for Network Pharmacology and Multi-Method Enrichment Analysis
Source: Int J Mol Sci. 2025 Nov 18;26(22):11147. doi: 10.3390/ijms262211147 (PMC12653797; doi:10.3390/ijms262211147)
Supplement: Supplementary file 1 [file ijms-26-11147-s001.zip › Supp Methods/Supplementary Method S1.pdf]

Supplementary Methods S1. Data validation specifications

Overview

This document provides detailed specifications for the data validation framework implemented in NeXus v1.2. The validation system ensures data integrity, format consistency, and relationship validity throughout the analysis pipeline.

1. Input data structure requirements
   1.1 File format specifications
   - Format: CSV (Comma-Separated Values)
   - Encoding: UTF-8
   - Maximum file size: 100 MB
   - Chunk processing size: 10,000 rows
   - Required columns: At minimum, a 'genes' column must be present
   - Optional columns: 'compounds', 'plants', 'relationships'

   1.2 Column name standards
   - Case insensitive: Column names converted to lowercase during processing
   - Whitespace handling: Leading and trailing whitespace stripped
   - Allowed characters: Letters, numbers, underscores
   - Reserved names: 'genes', 'gene', 'compound', 'compounds', 'plant', 'plants'

2. Gene identifier validation
   2.1 Format requirements
   Regular expression pattern:
   ^[A-Za-z0-9_-]+$

   Explanation: - Must start and end with alphanumeric character - Allowed characters: Letters (A-Z, a-z), numbers (0-9), underscore (_), hyphen (-) - No spaces or special characters - Case preserved during validation but converted to uppercase for analysis

   2.2 Length constraints
   - Minimum length: 2 characters
   - Maximum length: 50 characters
   - Rationale: Based on standard gene nomenclature across species databases

   2.3 Validation rules
   - Non-null requirement: Gene identifiers cannot be null or empty
   - Uniqueness: Duplicate gene identifiers flagged but not rejected (biological relevance)
   - Database compatibility: Validated against known gene identifier formats (NCBI, Ensembl, HGNC)
   - Character validation: Each character checked against allowed pattern

2.4 Error handling

Invalid gene identifier examples: - "GENE 1" (contains space) → Invalid - "G" (too short) → Invalid - "GENE@123" (contains special character @) → Invalid - "" (empty) → Invalid - NULL → Invalid

Valid gene identifier examples: - "TP53" → Valid - "BRCA1" → Valid - "ENSG00000141510" → Valid - "NM_000546" → Valid

3. Compound identifier validation

3.1 Format requirements

Regular expression pattern:

^[A-Za-z0-9\s-]+$

Explanation: - Allows spaces (compounds may have multi-word names) - Allowed characters: Letters, numbers, spaces, hyphens - No special characters except hyphen

3.2 Length constraints

- Minimum length: 3 characters
- Maximum length: 100 characters
- Rationale: Compound names can be longer than gene symbols

3.3 Validation rules

- Optional field: Compounds not required but recommended
- Whitespace normalization: Multiple spaces collapsed to single space
- Special character removal: Characters not in allowed pattern removed with warning
- Case handling: Compound names preserved in original case

3.4 Chemical identifier support

Supported formats: - Common names: “Curcumin”, “Resveratrol” - Chemical formulas: “C21H20O6” - Database IDs: “CHEMBL1234”, “CID123456” - SMILES notation: Not validated (treated as string)

4. Plant identifier validation

4.1 Format requirements

Regular expression pattern:

^[A-Za-z0-9\s._-]+$

Explanation: - Most permissive pattern (botanical names have varied formats) - Allows periods (for abbreviated taxonomy) - Allows spaces and underscores

4.2 Length constraints

- Minimum length: 1 character
- Maximum length: 150 characters

- Rationale: Full botanical names can be long (e.g., “Curcuma longa L.”)

4.3 Taxonomic name support

Supported formats: - Binomial nomenclature: “Curcuma longa” - Common names: “Turmeric” - Abbreviated: “C. longa” - With authority: “Curcuma longa L.”

4.4 Validation rules

- Optional field: Plants not required
- Title case conversion: Converted to title case for consistency
- Whitespace normalization: Multiple spaces collapsed
- Unicode support: Limited to Latin characters (ASCII extended)

5. Relationship validation

5.1 Compound-gene relationships

Validation checks: 1. Existence check: Both compound and gene must exist in respective lists 2. Maximum relationships per compound: 1,000 genes 3. Maximum relationships per gene: 1,000 compounds 4. Circular reference detection: None expected (bipartite graph)

Statistical metrics computed: - Average genes per compound - Average compounds per gene - Maximum degree (highest connectivity) - Minimum degree (lowest connectivity) - Isolated nodes (degree = 0)

5.2 Plant-compound relationships

Validation checks: 1. Existence check: Both plant and compound must exist 2. Maximum relationships per plant: 1,000 compounds 3. Maximum relationships per compound: 100 plants (multi-source compounds)

Metrics computed: - Shared compounds between plants (overlap analysis) - Plant-specific compounds (unique to one plant) - Average compounds per plant

6. Data quality metrics

6.1 Completeness metrics

```
completeness_metrics = {
    'total_rows': int,

    'valid_rows': int,

    'invalid_rows': int,

    'null_genes': int,

    'null_compounds': int,
```

```
        'null_plants': int,
        'completeness_percentage': float
    }
```

6.2 Validity metrics

```
validity_metrics = {
    'total_genes': int,
    'unique_genes': int,
    'invalid_genes': int,
    'valid_gene_percentage': float,

    'total_compounds': int,
    'unique_compounds': int,
    'invalid_compounds': int,
    'valid_compound_percentage': float,

    'total_plants': int,
    'unique_plants': int,
    'invalid_plants': int,
    'valid_plant_percentage': float
}
```

6.3 Relationship quality metrics

```
relationship_metrics = {
    'compound_gene_relationships': int,
    'plant_compound_relationships': int,
    'avg_genes_per_compound': float,
```

```
'max_genes_per_compound': int,

'avg_compounds_per_gene': float,

'orphan_genes': int,  # genes with no compounds

'orphan_compounds': int,  # compounds with no genes or plants

'relationship_density': float

}
```

7. Error threshold configuration
   7.1 Warning thresholds
   - Invalid entries > 5%: Warning issued
   - Null values > 10%: Warning issued
   - Orphan nodes > 20%: Warning issued

   7.2 Error thresholds (analysis blocked)
   - Invalid entries > 50%: Analysis cannot proceed
   - File size > 100 MB: File must be split
   - Rows > 1,000,000: Chunk processing required

   7.3 Quality gates
   Minimum requirements for analysis: - At least 1 valid gene - At least 50% of rows valid - File format parseable - Required columns present

8. Validation output
   8.1 Validation report structure

```
VALIDATION REPORT

================

Date: YYYY-MM-DD HH:MM:SS

File: input.csv


FILE VALIDATION:

- File size: X.XX MB

- Total rows: XXXX
```

- Format: Valid CSV ✓

GENE VALIDATION:

- Total genes: XXX
- Unique genes: XXX
- Invalid genes: XX (X.X%)
- Null genes: XX (X.X%)
- Status: PASS/WARN/FAIL

COMPOUND VALIDATION:

- Total compounds: XXX
- Unique compounds: XXX
- Invalid compounds: XX (X.X%)
- Status: PASS/WARN/FAIL

PLANT VALIDATION:

- Total plants: XXX
- Unique plants: XXX
- Invalid plants: XX (X.X%)
- Status: PASS/WARN/FAIL

RELATIONSHIP VALIDATION:

- Compound-gene relationships: XXXX
- Plant-compound relationships: XXX
- Orphan genes: XX
- Orphan compounds: XX
- Status: PASS/WARN/FAIL

OVERALL STATUS: PASS/WARN/FAIL

8.2 Error details

Each validation error includes: - Row number - Column name - Invalid value - Reason for failure - Suggested correction (if available)

9. Implementation notes

9.1 Performance optimization

- Chunk processing: Large files processed in 10,000-row chunks
- Parallel validation: Independent validations run concurrently
- Caching: Validation rules cached for repeated use
- Early termination: Critical errors stop validation immediately

9.2 Memory management

- Streaming: Files read in streaming mode to minimize memory
- Garbage collection: Explicit cleanup after chunk processing
- Maximum memory: ~500 MB for validation operations

9.3 Validation timing

Typical validation times: - 100 rows: <0.1 seconds - 1,000 rows: ~0.5 seconds - 10,000 rows: ~2 seconds - 100,000 rows: ~15 seconds

10. Example validation scenarios

Scenario 1: Perfect data

Input:

genes,compounds,plants

TP53,Curcumin,Curcuma longa

BRCA1,Resveratrol,Vitis vinifera

AKT1,Curcumin,Curcuma longa

Validation result: PASS (100% valid)

Scenario 2: Minor issues

Input:

genes,compounds,plants

TP53,Curcumin,Curcuma longa

BRCA1,,Vitis vinifera

AKT1,Resveratrol,

Validation result: WARN (null values present but <10%)

Scenario 3: Major issues

Input:

genes,compounds,plants

,Curcumin,Curcuma longa

GENE@123,Resveratrol,Vitis vinifera

A,Compound!Name,Plant123

Validation result: FAIL (>50% invalid entries)
