# Supplementary material for: NeXus: An Automated Platform for Network Pharmacology and Multi-Method Enrichment Analysis
Source: Int J Mol Sci. 2025 Nov 18;26(22):11147. doi: 10.3390/ijms262211147 (PMC12653797; doi:10.3390/ijms262211147)
Supplement: Supplementary file 1 [file ijms-26-11147-s001.zip › Supp Methods/Supplementary Method S2.pdf]

Supplementary Methods S2. Data preprocessing and standardization

Overview

This document details the data preprocessing and standardization procedures implemented in NeXus v1.2. These processes ensure data consistency, compatibility, and quality before network construction and enrichment analysis.

1. Column name standardization

   1.1 Normalization procedure

   Implementation:

   ```
   df.columns = df.columns.str.lower().str.strip()
   ```

   Processing steps: 1. Convert all column names to lowercase 2. Strip leading and trailing whitespace 3. Replace multiple spaces with single space 4. Remove special characters (except underscore)

   Examples: - " Genes " → "genes" - "Gene_Names" → "gene_names" - "COMPOUNDS" → "compounds" - "Plant Source" → "plant_source"

   1.2 Column mapping

   Standard column names recognized: - Gene columns: ['gene', 'genes', 'gene_name', 'gene_symbol', 'gene_id'] - Compound columns: ['compound', 'compounds', 'drug', 'chemical'] - Plant columns: ['plant', 'plants', 'herb', 'source']

   Automatic mapping: If non-standard column names detected, user prompted to map to standard names.

2. String data preprocessing

   2.1 Whitespace normalization

   Multiple Space Removal:

   ```
   df[col] = df[col].str.replace(r'\s+', ' ', regex=True)
   ```

   Processing: - Multiple consecutive spaces → single space - Tab characters → single space - Line breaks → single space - Leading/trailing whitespace → removed

   Example:

   "TP53   gene" → "TP53 gene"
   "BRCA1\t\tprotein" → "BRCA1 protein"
   " AKT1 " → "AKT1"

   2.2 Gene name standardization

   Uppercase Conversion:

df['genes'] = df['genes'].str.upper()

Rationale: - Gene symbols conventionally uppercase (HGNC guidelines) - Enables case-insensitive matching across databases - Prevents duplicate entries due to case differences

Examples: - "tp53" → "TP53" - "Brca1" → "BRCA1" - "akt1" → "AKT1"

2.3 Compound name cleaning

Special character removal:

df['compounds'] = df['compounds'].str.replace(r'[^\w\s-]', '', regex=True)

Characters removed: - Parentheses: () - Brackets: [] - Quotes: "' - Special symbols: @#$%^&*+=<>?/\ |

Characters preserved: - Letters: A-Z, a-z - Numbers: 0-9 - Spaces: - Hyphens: - - Underscores: _

Examples:

"Curcumin (natural)" → "Curcumin natural"
"Resveratrol-3-O-glucoside" → "Resveratrol-3-O-glucoside"
"Compound#123" → "Compound123"

2.4 Plant name formatting

Title case conversion:

df['plants'] = df['plants'].str.title()

Botanical Name Handling: - First word capitalized (Genus) - Second word lowercase (species) - Authority preserved if present

Examples:

"curcuma longa" → "Curcuma Longa"
"VITIS VINIFERA" → "Vitis Vinifera"
"panax ginseng" → "Panax Ginseng"

3. Null value handling

3.1 Null detection
Patterns recognized as null: - Python None - Pandas NaN - Empty strings: "" - Whitespace-only strings: "   " - Explicit null strings: "NULL", "NA", "N/A", "null", "na"

3.2 Mandatory field processing
Gene column (required):

df = df.dropna(subset=['genes'])
df = df[df['genes'].str.strip() != '']

Action: Rows with null/empty gene identifiers removed entirely

3.3 Optional field processing

Compound and Plant columns:

```
# Preserve rows, replace nulls with empty string
df['compounds'] = df['compounds'].fillna('')
df['plants'] = df['plants'].fillna('')
```

Action: Null values preserved (treated as missing data, not invalid)

3.4 Null value reporting

Statistics generated: - Total null values per column - Percentage of null values - Rows dropped due to mandatory field nulls - Warning if >10% null values in any column

4. Duplicate entry handling

4.1 Duplicate detection

Exact duplicates:

```
duplicates = df[df.duplicated(keep='first')]
```

Case-insensitive duplicates:

```
df_lower = df.applymap(lambda x: x.lower() if isinstance(x, str) else x)
duplicates_ci = df_lower[df_lower.duplicated(keep='first')]
```

4.2 Duplicate resolution strategy

Strategy 1: Keep First (default)

```
df = df.drop_duplicates(keep='first')
```

Strategy 2: Keep Last

```
df = df.drop_duplicates(keep='last')
```

Strategy 3: Remove All

```
df = df.drop_duplicates(keep=False)
```

4.3 Partial duplicates

Gene duplicates with different compounds: - Action: Keep all (biologically valid - one gene may interact with multiple compounds) - Log: Flagged for user review

Example:

```
TP53, Curcumin, Curcuma longa
TP53, Resveratrol, Vitis vinifera
```

Both rows kept (same gene, different compounds)

5. Data type conversion

5.1 String conversion

All data initially treated as strings to prevent premature type coercion.

```
df = df.astype(str)
```

Rationale: - Gene IDs may be numeric (e.g., "123456") but should be treated as strings - Prevents loss of leading zeros - Consistent handling across all fields

5.2 Categorical encoding (internal)

For performance optimization:

```
df['plants'] = df['plants'].astype('category')
df['compounds'] = df['compounds'].astype('category')
```

Benefits: - Reduced memory usage (factor encoding) - Faster grouping operations - Improved performance for large datasets

6. Relationship chain validation

6.1 Plant-compound-gene chain

Validation logic:

```
# Check if plant has compounds
plants_with_compounds = df.groupby('plants')['compounds'].nunique()

# Check if compounds have genes
compounds_with_genes = df.groupby('compounds')['genes'].nunique()

# Calculate chain completeness
chain_ratio = (plants_with_compounds > 0).sum() / len(plants_with_compounds)
```

6.2 Orphan node detection

Orphan genes: Genes without any compound associations

```
all_genes = set(df['genes'])
genes_with_compounds = set(df[df['compounds'] != '']['genes'])
orphan_genes = all_genes - genes_with_compounds
```

Orphan compounds: Compounds without gene or plant associations

```
compounds_with_genes = set(df[df['genes'] != '']['compounds'])
compounds_with_plants = set(df[df['plants'] != '']['compounds'])
complete_compounds = compounds_with_genes & compounds_with_plants
orphan_compounds = all_compounds - complete_compounds
```

6.3 Relationship statistics

Computed metrics:

```
relationship_stats = {
    'total_relationships': len(df),
    'unique_genes': df['genes'].nunique(),
    'unique_compounds': df['compounds'].nunique(),
    'unique_plants': df['plants'].nunique(),

    'avg_compounds_per_gene': df.groupby('genes')['compounds'].nunique().mean(),
    'max_compounds_per_gene': df.groupby('genes')['compounds'].nunique().max(),

    'avg_genes_per_compound': df.groupby('compounds')['genes'].nunique().mean(),
    'max_genes_per_compound': df.groupby('compounds')['genes'].nunique().max(),

    'avg_compounds_per_plant': df.groupby('plants')['compounds'].nunique().mean(),
    'max_compounds_per_plant': df.groupby('plants')['compounds'].nunique().max(),

    'shared_compounds': df.groupby('compounds')['plants'].nunique() > 1).sum(),
    'plant_specific_compounds': (df.groupby('compounds')['plants'].nunique() == 1).sum(),

    'orphan_genes': len(orphan_genes),
    'orphan_compounds': len(orphan_compounds)
}
```

7. Data quality filters

7.1 Minimum relationship threshold

Configuration: - Minimum genes per compound: 1 (default) - Minimum compounds per plant: 1 (default) - Configurable via config file

Filtering logic:

```
# Remove compounds with too few genes
compound_gene_counts = df.groupby('compounds')['genes'].nunique()
valid_compounds = compound_gene_counts[compound_gene_counts >= min_genes].index
df = df[df['compounds'].isin(valid_compounds)]
```

7.2 Maximum relationship threshold

Configuration: - Maximum genes per compound: 1,000 (prevents erroneous data) - Maximum compounds per gene: 1,000

Rationale: Values exceeding threshold likely indicate data errors or overly promiscuous hub nodes

8. Output format standardization

8.1 Cleaned data structure

Standard format:

```
genes,compounds,plants
TP53,Curcumin,Curcuma Longa
BRCA1,Resveratrol,Vitis Vinifera
AKT1,Curcumin,Curcuma Longa
```

Characteristics: - Consistent column names (lowercase) - Standardized case (genes uppercase, plants title case) - No null values in mandatory fields - No duplicate rows - Whitespace normalized

8.2 Preprocessing report

Saved as: preprocessing_report.txt

Contents:

```
PREPROCESSING REPORT
===================
Input file: input.csv
Processing date: YYYY-MM-DD HH:MM:SS

INITIAL DATA:
- Total rows: XXXX
- Total genes: XXX
- Total compounds: XXX
- Total plants: XXX

PREPROCESSING ACTIONS:
- Column name standardization: Applied
- Whitespace normalization: XX instances corrected
- Gene name uppercasing: XXX genes standardized
- Special character removal: XX characters removed
- Null value handling: XX rows removed (null genes)
- Duplicate removal: XX duplicates removed

FINAL DATA:
- Total rows: XXXX (XX% retained)
- Unique genes: XXX
- Unique compounds: XXX
- Unique plants: XXX

QUALITY METRICS:
- Orphan genes: XX (X.X%)
- Orphan compounds: XX (X.X%)
```

- Shared compounds: XX (X.X%)
- Average genes per compound: X.XX
- Average compounds per plant: X.XX

STATUS: SUCCESS/WARNING

9. Edge cases and special handling

9.1 Unicode characters

Policy: Limited support for ASCII extended characters only

Handling:

```
df = df.applymap(lambda x: x.encode('ascii', 'ignore').decode() if isinstance(x, str) else x)
```

9.2 Numeric gene IDs

Handling: Treated as strings, converted with leading zeros preserved

Example:

"00123" → "00123" (**not** converted to integer 123)

9.3 Mixed delimiter files

Detection and conversion: - Auto-detect delimiter (, ; \t) - Convert to standard CSV format - Warn user if non-standard delimiter detected

10. Performance metrics

10.1 Processing speed

Benchmark times (on standard hardware): - 100 rows: <0.05 seconds - 1,000 rows: ~0.2 seconds - 10,000 rows: ~1 second - 100,000 rows: ~8 seconds

10.2 Memory Usage

Input data: ~1 KB per row

Processing overhead: ~2x input size

Output data: ~0.8 KB per row (cleaned)
