# Supplementary material for: NeXus: An Automated Platform for Network Pharmacology and Multi-Method Enrichment Analysis
Source: Int J Mol Sci. 2025 Nov 18;26(22):11147. doi: 10.3390/ijms262211147 (PMC12653797; doi:10.3390/ijms262211147)
Supplement: Supplementary file 1 [file ijms-26-11147-s001.zip › Supp Methods/Supplementary Method S5.pdf]

Supplementary Methods S5. Enrichment analysis parameters and implementation

Overview

This document details the enrichment analysis implementation in NeXus v1.2, including three complementary methodologies: Over-Representation Analysis (ORA), Gene Set Enrichment Analysis (GSEA), and Gene Set Variation Analysis (GSVA). Complete parameter specifications, database versions, and statistical procedures are provided.

1. Enrichment analysis overview

1.1 Three-method approach

Method 1: Over-Representation Analysis (ORA) - Type: Threshold-based - Input: List of genes (binary: in/out of list) - Statistical test: Hypergeometric or Fisher's exact - Advantage: Simple, widely used, easy to interpret

Method 2: Gene Set Enrichment Analysis (GSEA) - Type: Threshold-free - Input: Ranked gene list (by expression, score, etc.) - Statistical test: Kolmogorov-Smirnov-like - Advantage: No arbitrary cutoff, detects coordinated changes

Method 3: Gene Set Variation Analysis (GSVA) - Type: Sample-wise - Input: Gene expression matrix - Statistical test: Non-parametric scoring - Advantage: Per-sample pathway scores, no phenotype labels needed

2. Database configuration

2.1 Biological databases used

Database	Version	Gene Sets	Update Frequency	Source
KEGG Pathways	2021	374 pathways	Quarterly	https://www.genome.jp/kegg/
GO Biological Process	2021-09	12,234 terms	Monthly	http://geneontology.org/
GO Molecular Function	2021-09	4,185 terms	Monthly	http://geneontology.org/
GO Cellular Component	2021-09	1,691 terms	Monthly	http://geneontology.org/
Reactome	2022	2,538 pathways	Quarterly	https://reactome.org/
WikiPathways	2021	752 pathways	Monthly	https://www.wikipathways.org/

2.2 Gene set size filters

Configuration:

```yaml
gene_set_filters:
  minimum_size: 15  # genes per set
  maximum_size: 500  # genes per set
  rationale: |
    - Sets <15: Too specific, low statistical power
    - Sets >500: Too general, difficult to interpret
```

Applied to all databases: - Remove gene sets with <15 genes - Remove gene sets with >500 genes - Log number of sets removed

2.3 Organism-Specific Configuration

Human (Homo sapiens): - KEGG organism code: hsa - GO organism: 9606 (NCBI Taxonomy ID) - Reactome species: Homo sapiens

Mouse (Mus musculus): - KEGG organism code: mmu - GO organism: 10090 - Configurable via config file

3. Over-representation analysis (ORA)

3.1 Statistical method

Hypergeometric test:

Formula for calculating enrichment p-value:

$$ $P(X \geq k) = \sum_{i=k}^{\min(n, K)} \frac{\binom{K}{i} \binom{N-K}{n-i}}{\binom{N}{n}} $$

$

Where: - N = total genes in background (universe) - K = genes in gene set (pathway) - n = genes in query list - k = overlap between query and gene set

Implementation:

```python
from scipy.stats import hypergeom

def ora_enrichment(gene_list, gene_set, background):
    """
    Perform ORA enrichment test
    """
    N = len(background)  # Total genes in universe
    K = len(gene_set & background)  # Gene set size
    n = len(gene_list & background)  # Query list size
    k = len(gene_list & gene_set)  # Overlap

    # Hypergeometric p-value
    p_value = hypergeom.sf(k-1, N, K, n)

    # Calculate enrichment ratio
```

```python
    expected = (n * K) / N
    fold_enrichment = k / expected if expected > 0 else 0

    return {
        'p_value': p_value,
        'overlap': k,
        'query_size': n,
        'gene_set_size': K,
        'fold_enrichment': fold_enrichment,
        'genes': list(gene_list & gene_set)
    }
```

3.2 Background gene universe

Default background:

```python
# All genes in database (union of all gene sets)
background = set()
for gene_set in all_gene_sets:
    background.update(gene_set)

# Typically 15,000-20,000 genes for human
```

Custom background: - User can provide custom background - Example: All genes assayed in experiment - Improves specificity for platform-specific analyses

3.3 Multiple testing correction

Benjamini-Hochberg (FDR):

```python
from statsmodels.stats.multitest import multipletests

# Adjust p-values
reject, pvals_corrected, _, _ = multipletests(
    pvals,
    alpha=0.05,
    method='fdr_bh'
)
```

Bonferroni (conservative):

```python
pvals_bonferroni = multipletests(pvals, method='bonferroni')[1]
```

Configuration:

```yaml
multiple_testing:
  primary_method: 'fdr_bh'  # Benjamini-Hochberg
  secondary_method: 'bonferroni'  # For comparison
  alpha: 0.05
```

4. Gene set enrichment analysis (GSEA)

4.1 Algorithm overview

GSEA Steps: 1. Rank genes by relevance score (expression, correlation, etc.) 2. For each gene set, calculate enrichment score (ES) 3. Permute gene labels to generate null distribution 4. Calculate normalized enrichment score (NES) and FDR

4.2 Enrichment score calculation

Running sum statistic:

$$ES(S) = \max_{1 \leq i \leq N} \left| \sum_{g_j \in S, j \leq i} \frac{|r_j|^p}{N_R} - \sum_{g_j \notin S, j \leq i} \frac{1}{N - N_R} \right|$$

$

Where: - S = gene set - N = total number of genes - N_R = sum of absolute correlation values for genes in S - r_j = ranking metric for gene j - p = weighting exponent (default = 1)

Implementation:

```python
import gseapy as gp

def run_gsea(gene_list, gene_sets, permutations=1000):
    """
    Run GSEA analysis using gseapy
    """
    # Format gene list as ranked list
    ranked_genes = pd.DataFrame({
        'gene': gene_list,
        'rank': range(len(gene_list))
    })

    # Run GSEA
    gsea_results = gp.prerank(
        rnk=ranked_genes,
        gene_sets=gene_sets,
        processes=4,
        permutation_num=permutations,
        outdir=None,
        format='png',
        seed=0
    )

    return gsea_results
```

4.3 Permutation strategy

Gene set permutation (default): - Permute gene set membership - Maintains gene-gene correlations - Appropriate when phenotype correlation is strong

Phenotype permutation: - Permute phenotype labels - Maintains gene set structure - Appropriate when gene sets are well-defined

Configuration:

```yaml
gsea:
  permutations: 1000
  permutation_type: 'gene_set'  # or 'phenotype'
  min_size: 15
  max_size: 500
  weighting_exponent: 1.0
  seed: 0  # for reproducibility
```

4.4 Normalized enrichment score (NES)

Normalization procedure:

NES = ES / mean(|ES_null|)

Where: - ES = observed enrichment score - ES_null = enrichment scores from permutations

Interpretation: - NES > 0: Enriched at top of ranked list - NES < 0: Enriched at bottom of ranked list - |NES| > 1.0: Potentially significant (check FDR)

4.5 Significance Thresholds

GSEA-specific thresholds:

```yaml
gsea_thresholds:
  nes_threshold: 1.0  # |NES| > 1.0
  pvalue_threshold: 0.05
  fdr_threshold: 0.25  # Standard GSEA threshold
```

Rationale for FDR < 0.25: - GSEA uses gene set permutations (conservative) - FDR < 0.25 recommended by Broad Institute - Balances sensitivity and specificity

5. Gene set variation analysis (GSVA)

5.1 Algorithm overview

GSVA generates sample-wise pathway scores: - Input: Gene expression matrix (genes × samples) - Output: Pathway score matrix (pathways × samples) - No phenotype labels required - Enables downstream comparative analysis

5.2 GSVA scoring method

Kernel density estimation:

For each sample j and gene set k:

$$ $GSVA_{jk} = \sum_{i=1}^{n} [I(g_i \in S_k) - I(g_i \notin S_k)] \cdot |r_i| $$

$

Where: - g_i = gene i - S_k = gene set k - r_i = rank-based score for gene i in sample j - I() = indicator function

Implementation:

```python
from gseapy import ssgsea

def run_gsva(expression_matrix, gene_sets):
    """
    Run GSVA analysis
    """
    gsva_results = ssgsea(
        data=expression_matrix,
        gene_sets=gene_sets,
        outdir=None,
        processes=4,
        permutation_num=0,  # GSVA doesn't use permutations
        min_size=15,
        max_size=500
    )

    return gsva_results
```

5.3 Kernel method selection

Available kernels: 1. Gaussian (default): - Smooth distribution - Robust to outliers - Recommended for most applications

Poisson:

For count data (RNA-seq)

Integer-based expression values

Bernoulli:

Binary presence/absence

For categorical data

Configuration:

```yaml
gsva:
  kernel_method: 'Gaussian'
  min_size: 15
  max_size: 500
```

```
  mx_diff: true  # Compute maximal difference
  abs_ranking: false
```

5.4 GSVA applications in NeXus

Use case in network pharmacology: - Generate pathway scores for each compound's gene targets - Compare pathway perturbation patterns across compounds - Identify complementary vs. redundant compounds

Implementation:

```
# For each compound, create pseudo-expression profile
# where target genes = 1, non-targets = 0
for compound in compounds:
    target_genes = get_target_genes(compound)
    pseudo_expression = create_binary_profile(target_genes, all_genes)
    gsva_scores[compound] = run_gsva(pseudo_expression, gene_sets)
```

6. Cross-method validation

6.1 Concordance analysis

Compare results across methods:

```
def compare_methods(ora_results, gsea_results, gsva_results):
    """
    Calculate concordance between methods
    """
    # Significant pathways from each method
    ora_sig = set(ora_results[ora_results['fdr'] < 0.05]['pathway'])
    gsea_sig = set(gsea_results[gsea_results['fdr'] < 0.25]['pathway'])
    gsva_sig = set(gsva_results[gsva_results['pvalue'] < 0.05]['pathway'])

    # Overlap analysis
    all_methods = ora_sig & gsea_sig & gsva_sig
    any_method = ora_sig | gsea_sig | gsva_sig

    concordance = len(all_methods) / len(any_method)

    return {
        'concordance': concordance,
        'ora_only': ora_sig - gsea_sig - gsva_sig,
        'gsea_only': gsea_sig - ora_sig - gsva_sig,
        'gsva_only': gsva_sig - ora_sig - gsea_sig,
        'all_methods': all_methods
    }
```

6.2 Method selection guidelines

When to use ORA: - Simple binary classification (disease vs. healthy) - Clear threshold for defining gene list - Need for easy interpretation

When to use GSEA: - Continuous ranking metric available - Want to avoid arbitrary thresholds - Interested in coordinated subtle changes

When to use GSVA: - No phenotype labels - Multiple samples/conditions - Want per-sample pathway scores

7. Result organization

7.1 Output structure

Per-entity enrichment:

```
enrichment_results/
├── global/
│   ├── ORA_all_genes.csv
│   ├── GSEA_all_genes.csv
│   └── GSVA_all_genes.csv
├── per_compound/
│   ├── compound1_ORA.csv
│   ├── compound1_GSEA.csv
│   ├── compound2_ORA.csv
│   └── ...
└── per_plant/
    ├── plant1_ORA.csv
    ├── plant1_GSEA.csv
    └── ...
```

7.2 Results table format

Standard columns:

```
pathway_id,pathway_name,database,p_value,fdr,genes_in_pathway,overlap_genes,fold_enrichment,nes,leading_edge_genes
```

Example:

```
hsa04010,MAPK signaling pathway,KEGG,1.3e-9,2.1e-7,295,23,4.2,2.1,"TP53,MAPK1,AKT1"
```

8. Performance pptimization

8.1 Parallel processing

Configuration:

```
# Use multiple cores for enrichment
enrichment_params = {
```

```
    'processes': 4,  # CPU cores
    'chunk_size': 100  # Gene sets per chunk
}
```

8.2 Caching strategy

Database caching:

```
# Cache gene sets to avoid repeated downloads
cache_dir = './.gsea_cache/'
gene_sets = load_gene_sets(database='KEGG', cache=True, cache_dir=cache_dir)
```

8.3 Processing Times

Benchmark times (3,000 gene query, 4 cores): - ORA: ~5 seconds - GSEA (1,000 permutations): ~45 seconds - GSVA: ~30 seconds - Total: ~1.5 minutes per entity

9. Quality control

9.1 Input validation

Gene identifier validation:

```
# Check gene identifier format
valid_genes = validate_gene_symbols(gene_list, organism='human')

# Map to official symbols if needed
mapped_genes = map_gene_identifiers(gene_list, from_type='ensembl', to_type='symbol')
```

9.2 Statistical validity checks

Checks performed: 1. Minimum gene list size (≥5 genes) 2. Overlap with database (≥50% genes recognized) 3. Multiple testing correction applied 4. P-value distribution check (avoid bimodal)

10. Reproducibility

10.1 Random seed configuration

All random operations seeded:

```
import random
import numpy as np

SEED = 42

random.seed(SEED)
np.random.seed(SEED)
```

```
# GSEA permutations
gsea_results = run_gsea(..., seed=SEED)
```

10.2 Version tracking

Database versions logged:


```json
{
  "analysis_date": "2025-10-28",
  "databases": {
    "KEGG": "2021",
    "GO": "2021-09",
    "Reactome": "2022"
  },
  "software_versions": {
    "gseapy": "1.0.4",
    "scipy": "1.7.3",
    "numpy": "1.21.5"
  }
}
```
