# Supplementary material for: NeXus: An Automated Platform for Network Pharmacology and Multi-Method Enrichment Analysis
Source: Int J Mol Sci. 2025 Nov 18;26(22):11147. doi: 10.3390/ijms262211147 (PMC12653797; doi:10.3390/ijms262211147)
Supplement: Supplementary file 1 [file ijms-26-11147-s001.zip › Supp Methods/Supplementary Method S6.pdf]

Supplementary Method S6. Visualization algorithms and rendering specifications

Overview

This document provides complete specifications for visualization generation in NeXus v1.2, including layout algorithms, color schemes, rendering parameters, and export formats. All figures are generated at 300 DPI for publication quality.

1. Visualization libraries

1.1 Core libraries

```
import matplotlib.pyplot as plt  # v3.5.0+
import seaborn as sns  # v0.11.0+
import networkx as nx  # v2.8.0+
import plotly.graph_objects as go  # v5.5.0+ (interactive)
from matplotlib import patches, collections
```

1.2 Configuration

```
# Global matplotlib settings
plt.rcParams['figure.dpi'] = 300
plt.rcParams['savefig.dpi'] = 300
plt.rcParams['font.family'] = 'sans-serif'
plt.rcParams['font.sans-serif'] = ['Arial', 'Helvetica']
plt.rcParams['font.size'] = 10
plt.rcParams['axes.linewidth'] = 1.5
plt.rcParams['xtick.major.width'] = 1.5
plt.rcParams['ytick.major.width'] = 1.5
```

2. Color palette design

2.1 Node type colors

Primary palette (consistent across all figures):

```
COLOR_PALETTE = {
    'gene': '#2E86AB',      # Blue
    'compound': '#06A77D',  # Green
    'plant': '#D62246',     # Red/Pink
}

# RGB values
RGB_VALUES = {
    'gene': (46, 134, 171),
    'compound': (6, 167, 125),
```

```
    'plant': (214, 34, 70)
}
```

Secondary colors (for gradients):

```
GRADIENT_COLORS = {
    'significance': {
        'high': '#8B0000',    # Dark red (p < 0.001)
        'medium': '#FF8C00',  # Orange (p < 0.01)
        'low': '#FFD700',     # Gold (p < 0.05)
        'ns': '#CCCCCC'       # Gray (not significant)
    },
    'enrichment': {
        'positive': '#D62246',  # Red
        'neutral': '#FFFFFF',   # White
        'negative': '#2E86AB'   # Blue
    }
}
```

2.2 Color blind accessibility

Palette tested against: - Deuteranopia (green-blind) - Protanopia (red-blind) - Tritanopia (blue-blind)

Tool used: Coblis Color Blindness Simulator

Verification: All critical distinctions remain visible

3. Network visualization

3.1 Layout algorithms

Spring Layout (Fruchterman-Reingold):

```
def spring_layout_custom(G, iterations=50, k=None):
    """
    Custom spring layout with adaptive parameters
    """
    n = G.number_of_nodes()

    # Optimal distance between nodes
    if k is None:
        k = 1 / np.sqrt(n)

    pos = nx.spring_layout(
        G,
        k=k,
        iterations=iterations,
        seed=42,  # Reproducibility
        scale=1.0,
```

```python
        center=(0, 0),
        weight='weight'
    )

    return pos
```

Algorithm: Force-directed layout - Attractive forces: Between connected nodes (edges) - Repulsive forces: Between all node pairs - Optimization: Minimize energy function

Parameters: - k: Optimal distance (1/√n for n nodes) - iterations: 50 (default), increase for large networks - seed: 42 (for reproducibility)

Circular Layout (for modules):

```python
def circular_layout_by_type(G, node_types):
    """
    Circular layout with node types in sectors
    """
    pos = {}
    angle_offset = 0

    for node_type in ['plant', 'compound', 'gene']:
        nodes = [n for n, t in node_types.items() if t == node_type]
        n_nodes = len(nodes)

        for i, node in enumerate(nodes):
            angle = angle_offset + (2 <i> np.pi </i> i / n_nodes)
            radius = 1.0
            pos[node] = (radius <i> np.cos(angle), radius </i> np.sin(angle))

        angle_offset += 2 <i> np.pi / 3  # 120° sectors

    return pos
```

3.2 Node rendering

Node size calculation:

```python
def calculate_node_sizes(G, base_size=500, scale_by='degree'):
    """
    Calculate node sizes based on network property
    """
    if scale_by == 'degree':
        degrees = dict(G.degree())
        max_degree = max(degrees.values())

        sizes = {
            node: base_size </i> (1 + 2 <i> deg / max_degree)
```

```
        for node, deg in degrees.items()
    }
elif scale_by == 'betweenness':
    betweenness = nx.betweenness_centrality(G)
    max_betweenness = max(betweenness.values())

    sizes = {
        node: base_size </i> (1 + 2 <i> bc / max_betweenness)
        for node, bc in betweenness.items()
    }
else:  # Fixed size
    sizes = {node: base_size for node in G.nodes()}

return sizes
```

Node shape specifications:

```
NODE_SHAPES = {
    'gene': 'o',       # Circle
    'compound': 's',  # Square
    'plant': 'D'       # Diamond
}
```

Node border:

```
node_border_params = {
    'linewidths': 1.5,
    'edgecolors': 'black'
}
```

3.3 Edge Rendering

Edge width calculation:

```
def calculate_edge_widths(G, base_width=1.0):
    """
    Calculate edge widths based on weight
    """
    weights = [G[u][v].get('weight', 1.0) for u, v in G.edges()]
    max_weight = max(weights)

    widths = [
        base_width </i> (0.5 + 1.5 <i> w / max_weight)
        for w in weights
    ]

    return widths
```

Edge opacity:

```
edge_alpha = 0.6  # Semi-transparent for overlapping edges
```

Edge color by type:

```
edge_colors = {
    'compound_gene': '#999999',  # Gray
    'plant_compound': '#CCCCCC'  # Light gray
}
```

3.4 Network Figure Assembly

Complete rendering pipeline:

```
def render_network(G, figsize=(12, 10), dpi=300):
    """
    Render complete network figure
    """
    fig, ax = plt.subplots(figsize=figsize, dpi=dpi)

    # Compute layout
    pos = spring_layout_custom(G)

    # Calculate sizes
    node_sizes = calculate_node_sizes(G, base_size=500)
    edge_widths = calculate_edge_widths(G)

    # Draw edges
    nx.draw_networkx_edges(
        G, pos, ax=ax,
        width=edge_widths,
        alpha=0.6,
        edge_color='gray'
    )

    # Draw nodes by type
    for node_type in ['gene', 'compound', 'plant']:
        nodelist = [n for n in G.nodes() if G.nodes[n]['node_type'] == node_type]

        nx.draw_networkx_nodes(
            G, pos, ax=ax,
            nodelist=nodelist,
            node_color=COLOR_PALETTE[node_type],
            node_size=[node_sizes[n] for n in nodelist],
            node_shape=NODE_SHAPES[node_type],
            linewidths=1.5,
            edgecolors='black',
            alpha=0.9
```

```
    )

    # Draw labels (top nodes only)
    top_nodes = sorted(G.degree, key=lambda x: x[1], reverse=True)[:20]
    labels = {node: G.nodes[node].get('label', node) for node, _ in top_nodes}

    nx.draw_networkx_labels(
        G, pos, labels, ax=ax,
        font_size=8,
        font_weight='bold'
    )

    # Formatting
    ax.axis('off')
    ax.margins(0.1)
    plt.tight_layout()

    return fig, ax
```

4. Enrichment visualization

4.1 Bar plot specifications

Horizontal bar plot:

```
def plot_enrichment_bars(enrichment_df, top_n=15, figsize=(8, 10)):
    """
    Create horizontal bar plot for enrichment results
    """
    # Sort by adjusted p-value
    plot_df = enrichment_df.nsmallest(top_n, 'fdr')

    fig, ax = plt.subplots(figsize=figsize, dpi=300)

    # Calculate bar colors based on significance
    colors = []
    for pval in plot_df['fdr']:
        if pval < 0.001:
            colors.append('#8B0000')
        elif pval < 0.01:
            colors.append('#FF8C00')
        elif pval < 0.05:
            colors.append('#FFD700')
        else:
            colors.append('#CCCCCC')

    # Create bars
```

```
    y_pos = np.arange(len(plot_df))
    bars = ax.barh(
        y_pos,
        -np.log10(plot_df['fdr']),
        color=colors,
        edgecolor='black',
        linewidth=1.5
    )

    # Significance threshold line
    ax.axvline(
        x=-np.log10(0.05),
        color='red',
        linestyle='--',
        linewidth=2,
        label='FDR = 0.05'
    )

    # Formatting
    ax.set_yticks(y_pos)
    ax.set_yticklabels(plot_df['pathway_name'], fontsize=10)
    ax.set_xlabel('-log10(FDR)', fontsize=12, fontweight='bold')
    ax.set_title('Enrichment Analysis', fontsize=14, fontweight='bold')
    ax.legend()
    ax.spines['top'].set_visible(False)
    ax.spines['right'].set_visible(False)

    plt.tight_layout()
    return fig, ax
```

4.2 Bubble plot specifications

Multi-dimensional bubble plot:

```
def plot_enrichment_bubble(enrichment_df, top_n=30, figsize=(12, 10)):
    """
    Create bubble plot: size=gene ratio, color=p-value, x=fold enrichment
    """
    plot_df = enrichment_df.nsmallest(top_n, 'fdr')

    fig, ax = plt.subplots(figsize=figsize, dpi=300)

    # Bubble sizes (gene ratio)
    sizes = (plot_df['overlap'] / plot_df['gene_set_size'] </i> 1000)

    # Bubble colors (-log10 p-value)
```

```
    colors = -np.log10(plot_df['fdr'])

    # Scatter plot
    scatter = ax.scatter(
        plot_df['fold_enrichment'],
        range(len(plot_df)),
        s=sizes,
        c=colors,
        cmap='YlOrRd',
        edgecolors='black',
        linewidths=1.5,
        alpha=0.8
    )

    # Color bar
    cbar = plt.colorbar(scatter, ax=ax)
    cbar.set_label('-log10(FDR)', fontsize=12, fontweight='bold')

    # Formatting
    ax.set_yticks(range(len(plot_df)))
    ax.set_yticklabels(plot_df['pathway_name'], fontsize=9)
    ax.set_xlabel('Fold Enrichment', fontsize=12, fontweight='bold')
    ax.set_title('Pathway Enrichment', fontsize=14, fontweight='bold')
    ax.grid(alpha=0.3, linestyle='--')
    ax.spines['top'].set_visible(False)
    ax.spines['right'].set_visible(False)

    plt.tight_layout()
    return fig, ax
```

4.3 Heatmap specifications

Gene-term association heatmap:

```
def plot_enrichment_heatmap(gene_term_matrix, figsize=(12, 10)):
    """
    Create heatmap showing gene-term associations
    """
    fig, ax = plt.subplots(figsize=figsize, dpi=300)

    # Create heatmap
    sns.heatmap(
        gene_term_matrix,
        cmap='RdYlBu_r',
        center=0,
        cbar_kws={'label': 'Association Score'},
```

```
        linewidths=0.5,
        linecolor='gray',
        square=False,
        ax=ax
    )

    # Formatting
    ax.set_xlabel('Pathways', fontsize=12, fontweight='bold')
    ax.set_ylabel('Genes', fontsize=12, fontweight='bold')
    ax.set_title('Gene-Pathway Association Matrix', fontsize=14, fontweight='bold')

    plt.xticks(rotation=45, ha='right')
    plt.yticks(rotation=0)
    plt.tight_layout()

    return fig, ax
```

5. Statistical plots

5.1 Degree distribution

Log-log plot with power-law fit:

```
def plot_degree_distribution(G, figsize=(8, 6)):
    """
    Plot degree distribution with power-law fit
    """
    degrees = [d for n, d in G.degree()]
    degree_count = Counter(degrees)

    fig, ax = plt.subplots(figsize=figsize, dpi=300)

    # Plot distribution
    x = list(degree_count.keys())
    y = list(degree_count.values())

    ax.loglog(x, y, 'bo', markersize=8, alpha=0.7, label='Observed')

    # Power-law fit
    from scipy.optimize import curve_fit

    def powerlaw(k, gamma, C):
        return C <i> k<b>(-gamma)

    popt, _ = curve_fit(powerlaw, x, y, p0=[2.0, 1.0])
    gamma, C = popt
```

```
    x_fit = np.logspace(np.log10(min(x)), np.log10(max(x)), 100)
    y_fit = powerlaw(x_fit, gamma, C)

    ax.loglog(x_fit, y_fit, 'r-', linewidth=2,
             label=f'Power law fit: γ={gamma:.2f}')

    # Formatting
    ax.set_xlabel('Degree (k)', fontsize=12, fontweight='bold')
    ax.set_ylabel('Frequency P(k)', fontsize=12, fontweight='bold')
    ax.set_title('Degree Distribution', fontsize=14, fontweight='bold')
    ax.legend()
    ax.grid(alpha=0.3)

    plt.tight_layout()
    return fig, ax
```

5.2 Box plots

Centrality measure comparison:

```
def plot_centrality_boxplots(centrality_dict, figsize=(10, 6)):
    """
    Create box plots comparing centrality measures by node type
    """
    fig, ax = plt.subplots(figsize=figsize, dpi=300)

    # Prepare data
    data = []
    labels = []
    for node_type in ['gene', 'compound', 'plant']:
        values = [
            centrality_dict[node]
            for node in centrality_dict
            if node.startswith(node_type)
        ]
        data.append(values)
        labels.append(node_type.capitalize())

    # Create box plot
    bp = ax.boxplot(
        data,
        labels=labels,
        patch_artist=True,
        notch=True,
        widths=0.6
    )
```

```
    # Color boxes
    colors = [COLOR_PALETTE['gene'], COLOR_PALETTE['compound'], COLOR_PALETTE['plant']]
    for patch, color in zip(bp['boxes'], colors):
        patch.set_facecolor(color)
        patch.set_alpha(0.7)

    # Formatting
    ax.set_ylabel('Centrality', fontsize=12, fontweight='bold')
    ax.set_title('Centrality by Node Type', fontsize=14, fontweight='bold')
    ax.grid(axis='y', alpha=0.3)

    plt.tight_layout()
    return fig, ax
```

6. Multi-panel figures

6.1 Figure grid layout

Creating composite figures:

```
def create_multipanel_figure(nrows=2, ncols=2, figsize=(16, 14)):
    """
    Create multi-panel figure with labeled subplots
    """
    fig, axes = plt.subplots(nrows, ncols, figsize=figsize, dpi=300)

    # Add panel labels (A, B, C, D, ...)
    labels = ['A', 'B', 'C', 'D', 'E', 'F']
    for idx, ax in enumerate(axes.flat):
        if idx < len(labels):
            ax.text(
                -0.1, 1.1, labels[idx],
                transform=ax.transAxes,
                fontsize=16,
                fontweight='bold',
                va='top', ha='right'
            )

    plt.tight_layout()
    return fig, axes
```

7. Export specifications

7.1 File formats

PNG (primary):

```python
fig.savefig(
    'output.png',
    dpi=300,
    bbox_inches='tight',
    facecolor='white',
    edgecolor='none',
    transparent=False
)
```

PDF (vector):

```python
fig.savefig(
    'output.pdf',
    format='pdf',
    bbox_inches='tight',
    dpi=300
)
```

SVG (editable):

```python
fig.savefig(
    'output.svg',
    format='svg',
    bbox_inches='tight'
)
```

7.2 Resolution standards

DPI specifications: - Publication quality: 300 DPI (required) - Screen display: 150 DPI - Draft/preview: 72 DPI

File size optimization:

```python
from PIL import Image

def optimize_png(filename, max_size_mb=10):
    """
    Optimize PNG file size while maintaining quality
    """
    img = Image.open(filename)

    # Convert RGBA to RGB if needed
    if img.mode == 'RGBA':
        background = Image.new('RGB', img.size, (255, 255, 255))
        background.paste(img, mask=img.split()[3])
        img = background

    # Save with optimization
    img.save(
```

```
        filename,
        'PNG',
        optimize=True,
        quality=95
    )
```

8. Interactive visualizations

8.1 Plotly network graph

Interactive network (HTML export):

```
def create_interactive_network(G):
    """
    Create interactive Plotly network
    """
    import plotly.graph_objects as go

    pos = spring_layout_custom(G)

    # Edge traces
    edge_trace = go.Scatter(
        x=[], y=[],
        line=dict(width=0.5, color='#888'),
        hoverinfo='none',
        mode='lines'
    )

    for edge in G.edges():
        x0, y0 = pos[edge[0]]
        x1, y1 = pos[edge[1]]
        edge_trace['x'] += (x0, x1, None)
        edge_trace['y'] += (y0, y1, None)

    # Node traces
    node_trace = go.Scatter(
        x=[pos[node][0] for node in G.nodes()],
        y=[pos[node][1] for node in G.nodes()],
        mode='markers',
        hoverinfo='text',
        marker=dict(
            showscale=True,
            colorscale='YlOrRd',
            size=10,
            colorbar=dict(thickness=15, title='Degree', xanchor='left')
        )
    )
```

```
# Create figure
fig = go.Figure(
    data=[edge_trace, node_trace],
    layout=go.Layout(
        showlegend=False,
        hovermode='closest',
        xaxis=dict(showgrid=False, zeroline=False, showticklabels=False),
        yaxis=dict(showgrid=False, zeroline=False, showticklabels=False)
    )
)

return fig
```
