# Supplementary material for: NeXus: An Automated Platform for Network Pharmacology and Multi-Method Enrichment Analysis
Source: Int J Mol Sci. 2025 Nov 18;26(22):11147. doi: 10.3390/ijms262211147 (PMC12653797; doi:10.3390/ijms262211147)
Supplement: Supplementary file 1 [file ijms-26-11147-s001.zip › Supp Methods/Supplementary Method S7.pdf]

Supplementary Method S7. Output management and data organization

Overview

This document specifies the output management system in NeXus v1.2, including directory architecture, file naming conventions, data serialization formats, and provenance tracking for complete reproducibility.

1. Directory architecture

1.1 Root output structure

Standard directory tree:

```
NEXUS_OUTPUT_YYYYMMDD_HHMMSS/
├── 1_network/
│   ├── network.gexf
│   ├── network.json
│   ├── adjacency_matrix.csv
│   └── network_metrics.json
├── 2_figures/
│   ├── network/
│   ├── enrichment/
│   └── visualization/
├── 3_gene_selection/
│   ├── gene_lists/
│   ├── selection_reports/
│   └── metadata/
├── 4_enrichment/
│   ├── ORA/
│   ├── GSEA/
│   ├── GSVA/
│   └── combined_results/
├── logs/
│   ├── analysis.log
│   ├── errors.log
│   └── warnings.log
├── config/
│   ├── analysis_config.yaml
│   └── parameters.json
└── ANALYSIS_REPORT.md
```

Naming convention: - Timestamp format: YYYYMMDD_HHMMSS (e.g., 20251028_143052) - Ensures unique directories for each analysis run - Allows chronological sorting

1.2 Network directory (1_network/)

Contents:

```
1_network/
├── network.gexf            # Primary network file (Gephi format)
├── network.json            # JSON format (web-compatible)
├── adjacency_matrix.csv     # Matrix format
├── edge_list.csv           # Simple edge list
├── node_attributes.csv      # Node metadata
├── network_metrics.json     # Computed metrics
└──── README.txt             # Directory description
```

File descriptions:

network.gexf: - Format: Graph Exchange XML Format - Use: Import to Gephi, Cytoscape - Preserves: All node/edge attributes - Size: ~1-10 MB (typical)

network.json: - Format: Node-link format JSON - Use: Web visualizations, D3.js - Structure:


```json
{
  "nodes": [
    {"id": "gene_TP53", "type": "gene", "label": "TP53", "degree": 23},
    {"id": "compound_Curcumin", "type": "compound", "label": "Curcumin", "degree": 18}
  ],
  "links": [
    {"source": "gene_TP53", "target": "compound_Curcumin", "weight": 1.0}
  ]
}
```


network_metrics.json: - Contains: All computed topological metrics - Structure:


```json
{
  "global_metrics": {
    "n_nodes": 143,
    "n_edges": 1033,
    "density": 0.1017,
    "avg_clustering": 0.374,
    "avg_path_length": 3.2,
    "modularity": 0.428
  },
  "centrality": {
    "gene_TP53": {"degree": 0.23, "betweenness": 0.18, "closeness": 0.42},
    "compound_Curcumin": {"degree": 0.19, "betweenness": 0.12, "closeness": 0.38}
  },
  "communities": {
    "n_communities": 6,
    "sizes": [38, 32, 28, 22, 18, 14],
```

"assignments": {"gene_TP53": 0, "compound_Curcumin": 0}
  }
}

1.3 Figures directory (2_figures/)

Subdirectory structure:

```
2_figures/
├── network/
│   ├── Network_Degree_Distribution.png (300 DPI)
│   ├── Network_Node_Type_Distribution.png (300 DPI)
│   ├── Network_Clustering_Distribution.png (300 DPI)
│   └── Network_Communities.png (300 DPI)
├── enrichment/
│   ├── Enrichment_Bubble_Plot.png (300 DPI)
│   ├── Enrichment_BarPlots_ByDatabase.png (300 DPI)
│   ├── Enrichment_Heatmap_KEGG.png (300 DPI)
│   ├── Enrichment_Heatmap_GO_BP.png (300 DPI)
│   └── per_entity/
│       ├── compound_Curcumin_enrichment.png
│       └── plant_Curcuma_longa_enrichment.png
└── visualization/
    ├── Figure1_Network_Overview.png (300 DPI)
    ├── Figure2_Statistical_Analysis.png (300 DPI)
    ├── Figure3_Compound_Target_Matrix.png (300 DPI)
    ├── Figure4_Network_Topology.png (300 DPI)
    └── Figure5_Hierarchical_Analysis.png (300 DPI)
```

File naming rules: - Descriptive names: Indicate content clearly - Underscores: Word separators (not hyphens or spaces) - Capital letters: For major words - Suffix: Always .png at 300 DPI

1.4 Enrichment directory (4_enrichment/)

Method-specific subdirectories:

```
4_enrichment/
├── ORA/
│   ├── global_ORA_results.csv
│   ├── global_ORA_KEGG.csv
│   ├── global_ORA_GO_BP.csv
│   ├── global_ORA_GO_MF.csv
│   ├── global_ORA_GO_CC.csv
│   ├── per_compound/
│   │   ├── compound1_ORA_KEGG.csv
```

```
│   │   └── compound2_ORA_KEGG.csv
│   └── per_plant/
│       ├── plant1_ORA_KEGG.csv
│       └── plant2_ORA_KEGG.csv
├── GSEA/
│   ├── global_GSEA_results.csv
│   ├── enrichment_plots/
│   └── leading_edge_genes/
├── GSVA/
│   ├── pathway_scores_matrix.csv
│   ├── sample_enrichment_profiles.csv
│   └── heatmaps/
└── combined_results/
    ├── method_comparison.csv
    ├── consensus_pathways.csv
    └── concordance_analysis.txt
```

CSV format standardization:

```
pathway_id,pathway_name,database,p_value,adjusted_p_value,fold_enrichment,overlap,query_size,pathway_size,genes
hsa04010,MAPK signaling pathway,KEGG,1.3e-9,2.1e-7,4.2,23,111,295,"TP53,MAPK1,AKT1,..."
```

2. File naming conventions

2.1 Network files

Format: network.[format]

Examples: - network.gexf - network.json - network_filtered.gexf (if filtered version exists)

2.2 Figure files

Format: [Category]_[Description].[ext]

Examples: - Network_Degree_Distribution.png - Enrichment_Bubble_Plot.png - Figure1_Network_Overview.png

Rules: - Start with category (Network, Enrichment, Figure) - Use descriptive middle section - Extension indicates format (.png, .pdf, .svg)

2.3 Data files

Format: [entity]_[method]_[database].[ext]

Examples: - global_ORA_KEGG.csv - compound1_GSEA_GO_BP.csv - plant_Curcuma_longa_ORA_KEGG.csv

Rules: - Entity: global, compound, plant, gene - Method: ORA, GSEA, GSVA - Database: KEGG, GO_BP, GO_MF, GO_CC - Always lowercase with underscores

2.4 Log files

Format: [type].log

Examples: - analysis.log - Main analysis log - errors.log - Error messages only - warnings.log - Warning messages only - debug.log - Debug information

3. Data serialization formats

3.1 Network serialization

GEXF (XML-based):

```xml
<?xml version="1.0" encoding="UTF-8"?>
<gexf xmlns="http://www.gexf.net/1.2draft">
  <graph mode="static" defaultedgetype="undirected">
    <attributes class="node">
      <attribute id="0" title="node_type" type="string"/>
      <attribute id="1" title="label" type="string"/>
      <attribute id="2" title="degree" type="integer"/>
    </attributes>
    <nodes>
      <node id="gene_TP53" label="TP53">
        <attvalues>
          <attvalue for="0" value="gene"/>
          <attvalue for="1" value="TP53"/>
          <attvalue for="2" value="23"/>
        </attvalues>
      </node>
    </nodes>
    <edges>
      <edge id="0" source="gene_TP53" target="compound_Curcumin" weight="1.0"/>
    </edges>
  </graph>
</gexf>
```

JSON serialization:

```python
import json
import networkx as nx
from networkx.readwrite import json_graph

def serialize_network_json(G, filename):
```

```python
    """
    Serialize network to JSON format
    """
    data = json_graph.node_link_data(G)

    with open(filename, 'w') as f:
        json.dump(data, f, indent=2)
```

3.2 Metrics serialization

JSON format (preferred for metrics):

```python
import json

def serialize_metrics(metrics, filename):
    """
    Serialize metrics dictionary to JSON
    """
    # Convert numpy types to native Python types
    def convert_types(obj):
        if isinstance(obj, np.integer):
            return int(obj)
        elif isinstance(obj, np.floating):
            return float(obj)
        elif isinstance(obj, np.ndarray):
            return obj.tolist()
        else:
            return obj

    metrics_converted = {
        k: convert_types(v) for k, v in metrics.items()
    }

    with open(filename, 'w') as f:
        json.dump(metrics_converted, f, indent=2, sort_keys=True)
```

3.3 Enrichment results serialization

CSV format (primary):

```python
import pandas as pd

def serialize_enrichment_results(results_df, filename):
    """
```

```python
    Save enrichment results to CSV
    """
    # Standardize column order
    column_order = [
        'pathway_id', 'pathway_name', 'database',
        'p_value', 'adjusted_p_value', 'fold_enrichment',
        'overlap', 'query_size', 'pathway_size', 'genes'
    ]

    # Reorder columns if all present
    if all(col in results_df.columns for col in column_order):
        results_df = results_df[column_order]

    # Save with standardized options
    results_df.to_csv(
        filename,
        index=False,
        float_format='%.2e',  # Scientific notation for p-values
        encoding='utf-8'
    )
```

4. Provenance tracking

4.1 Analysis configuration

Saved as: config/analysis_config.yaml


```yaml
analysis_metadata:
  analysis_id: "NEXUS_20251028_143052"
  start_time: "2025-10-28 14:30:52"
  end_time: "2025-10-28 14:35:47"
  duration_seconds: 295
  nexus_version: "1.2.0"
  python_version: "3.9.7"

input_data:
  file: "input.csv"
  file_size_bytes: 15842
  md5_hash: "a1b2c3d4e5f6..."
  n_rows: 234
  n_genes: 111
  n_compounds: 32
  n_plants: 3
```

```yaml
processing_parameters:
  validation:
    max_file_size_mb: 100
    min_genes_per_compound: 1
  network_construction:
    layout_algorithm: "spring"
    layout_iterations: 50
    min_edge_weight: 0.1
  enrichment_analysis:
    methods: ["ORA", "GSEA", "GSVA"]
    databases: ["KEGG", "GO_BP", "GO_MF", "GO_CC"]
    ora_p_threshold: 0.05
    ora_correction_method: "fdr_bh"
    gsea_permutations: 1000
    gsea_fdr_threshold: 0.25
  visualization:
    figure_dpi: 300
    figure_format: "png"

software_versions:
  networkx: "2.8.0"
  pandas: "1.4.2"
  matplotlib: "3.5.1"
  seaborn: "0.11.2"
  gseapy: "1.0.4"
  scipy: "1.7.3"
  numpy: "1.21.5"

database_versions:
  KEGG: "2021"
  GO: "2021-09"
  Reactome: "2022"
  WikiPathways: "2021"
```


4.2 Logging system

Log file format:

```
2025-10-28 14:30:52,123 - INFO - NeXus v1.2 - Analysis started
2025-10-28 14:30:52,234 - INFO - Input file: input.csv (15.5 KB, 234 rows)
2025-10-28 14:30:53,456 - INFO - Validation complete: 111 genes, 32 compounds, 3 plants
2025-10-28 14:30:54,567 - INFO - Network construction: 143 nodes, 1033 edges
2025-10-28 14:30:55,678 - INFO - Centrality computation: 4 metrics calculated
2025-10-28 14:31:02,789 - INFO - Community detection: 6 communities found
```

2025-10-28 14:31:15,890 - INFO - ORA enrichment: 42 significant pathways (FDR < 0.05)
2025-10-28 14:32:01,123 - INFO - GSEA enrichment: 38 significant pathways (FDR < 0.25)
2025-10-28 14:32:45,234 - INFO - GSVA scores calculated
2025-10-28 14:33:20,345 - INFO - Visualization: 15 figures generated (300 DPI)
2025-10-28 14:35:47,456 - INFO - Analysis complete (295 seconds)
2025-10-28 14:35:47,567 - INFO - Output saved to: NEXUS_20251028_143052/

Logging configuration:

```python
import logging
from logging.handlers import RotatingFileHandler

def setup_logging(output_dir):
    """
    Configure logging system
    """
    log_dir = os.path.join(output_dir, 'logs')
    os.makedirs(log_dir, exist_ok=True)

    # Main log file
    logger = logging.getLogger('nexus')
    logger.setLevel(logging.DEBUG)

    # File handler (all messages)
    fh = RotatingFileHandler(
        os.path.join(log_dir, 'analysis.log'),
        maxBytes=10<i>1024</i>1024,  # 10 MB
        backupCount=5
    )
    fh.setLevel(logging.DEBUG)

    # Console handler (INFO and above)
    ch = logging.StreamHandler()
    ch.setLevel(logging.INFO)

    # Format
    formatter = logging.Formatter(
        '%(asctime)s - %(name)s - %(levelname)s - %(message)s'
    )
    fh.setFormatter(formatter)
    ch.setFormatter(formatter)

    logger.addHandler(fh)
    logger.addHandler(ch)
```

```
    return logger
```

5. Report generation

5.1 Analysis report structure

Saved as: ANALYSIS_REPORT.md

```
# NeXus Analysis Report

<b>Analysis ID:</b> NEXUS_20251028_143052
<b>Date:</b> 2025-10-28 14:35:47
<b>Duration:</b> 295 seconds (4 min 55 sec)

---

## Input Summary

- <b>File:</b> input.csv
- <b>Size:</b> 15.5 KB
- <b>Genes:</b> 111 unique
- <b>Compounds:</b> 32 unique
- <b>Plants:</b> 3 unique
- <b>Relationships:</b> 234 entries

---

## Network Analysis

### Network Properties
- <b>Nodes:</b> 143 (108 genes, 32 compounds, 3 plants)
- <b>Edges:</b> 1,033
- <b>Density:</b> 0.1017
- <b>Average clustering coefficient:</b> 0.374
- <b>Average path length:</b> 3.2
- <b>Number of components:</b> 1 (fully connected)

### Community Structure
- <b>Number of communities:</b> 6
- <b>Modularity (Q):</b> 0.428
- <b>Community sizes:</b> [38, 32, 28, 22, 18, 14]

### Hub Nodes (Top 10 by degree)
```

1. TP53 (gene) - degree: 23
2. AKT1 (gene) - degree: 21
3. MAPK1 (gene) - degree: 20
...

Enrichment Analysis

ORA Results
- Significant pathways: 42 (FDR < 0.05)
- Top pathway: MAPK signaling (p = 1.3e-9, fold enrichment = 4.2)

GSEA Results
- Significant pathways: 38 (FDR < 0.25)
- Top pathway: TNF signaling (NES = 2.4, p = 2.7e-8)

Method Concordance
- ORA ∩ GSEA: 28 pathways (66% overlap)
- Unique to ORA: 14 pathways
- Unique to GSEA: 10 pathways

Output Files

Network Files
- ``1_network/network.gexf`` - Primary network (Gephi format)
- ``1_network/network.json`` - JSON format
- ``1_network/network_metrics.json`` - Computed metrics

Figures (300 DPI)
- ``2_figures/visualization/`` - 5 publication figures
- ``2_figures/network/`` - 4 network analysis plots
- ``2_figures/enrichment/`` - 6 enrichment visualizations

Enrichment Results
- ``4_enrichment/ORA/`` - ORA results per database
- ``4_enrichment/GSEA/`` - GSEA results
- ``4_enrichment/GSVA/`` - GSVA pathway scores

Analysis completed successfully.

6. Data backup and archiving

6.1 Archive creation

Create compressed archive:

```
import shutil
import os

def create_archive(output_dir):
    """
    Create compressed archive of analysis output
    """
    archive_name = f"{os.path.basename(output_dir)}.zip"

    shutil.make_archive(
        base_name=output_dir,
        format='zip',
        root_dir=os.path.dirname(output_dir),
        base_dir=os.path.basename(output_dir)
    )

    return archive_name
```

6.2 Checksums

Generate MD5 checksums:

```
import hashlib

def generate_checksums(output_dir):
    """
    Generate MD5 checksums for all files
    """
    checksums = {}

    for root, dirs, files in os.walk(output_dir):
        for file in files:
            filepath = os.path.join(root, file)
            relative_path = os.path.relpath(filepath, output_dir)

            with open(filepath, 'rb') as f:
```

```python
            checksum = hashlib.md5(f.read()).hexdigest()

        checksums[relative_path] = checksum

    # Save checksums
    with open(os.path.join(output_dir, 'checksums.txt'), 'w') as f:
        for path, checksum in sorted(checksums.items()):
            f.write(f"{checksum}  {path}\n")
```

7. Performance metrics

7.1 File size estimates

Typical file sizes: - Network GEXF: 1-5 MB - Network JSON: 2-10 MB - Network metrics JSON: 100-500 KB - Figure PNG (300 DPI): 200 KB - 1 MB - Enrichment CSV (per database): 50-200 KB - Log files: 100 KB - 2 MB

Total output size: 20-100 MB (typical)

7.2 Write performance

Optimization: - Asynchronous I/O for large files - Buffered writes for CSVs - Compressed archives for storage
