# Supplementary material for: NeXus: An Automated Platform for Network Pharmacology and Multi-Method Enrichment Analysis
Source: Int J Mol Sci. 2025 Nov 18;26(22):11147. doi: 10.3390/ijms262211147 (PMC12653797; doi:10.3390/ijms262211147)
Supplement: Supplementary file 1 [file ijms-26-11147-s001.zip › Supp Methods/Supplementary Method S8.pdf]

Supplementary Method S8. Statistical methods and validation protocols

Overview

This document provides complete statistical methods, significance testing procedures, and validation protocols implemented in NeXus v1.2. All statistical procedures follow established conventions and best practices for network pharmacology and enrichment analysis.

1. Statistical significance testing

1.1 P-value calculation methods

Hypergeometric test (for ORA):

Formula:

$$P(X \geq k) = \sum_{i=k}^{\min(n,K)} \frac{\binom{K}{i}\binom{N-K}{n-i}}{\binom{N}{n}}$$

Implementation:

```python
from scipy.stats import hypergeom

def calculate_hypergeometric_pvalue(k, n, K, N):
    """
    Calculate hypergeometric p-value

    Parameters:
    k: overlap size (query ∩ gene set)
    n: query size
    K: gene set size
    N: background universe size

    Returns:
    p_value: probability of observing k or more overlaps by chance
    """
    p_value = hypergeom.sf(k-1, N, K, n)
    return p_value
```

Kolmogorov-Smirnov Test (for GSEA):

Formula (two-sample KS statistic):

$$D_{n,m} = \sup_x |F_n(x) - F_m(x)|$$

Where: - F_n = empirical distribution function of sample 1 - F_m = empirical distribution function of sample 2

Implementation:

```python
from scipy.stats import ks_2samp

def calculate_ks_pvalue(genes_in_set, genes_not_in_set):
    """
    Calculate Kolmogorov-Smirnov p-value for GSEA

    Parameters:
    genes_in_set: ranks of genes in pathway
    genes_not_in_set: ranks of genes not in pathway

    Returns:
    statistic, p_value
    """
    statistic, p_value = ks_2samp(genes_in_set, genes_not_in_set)
    return statistic, p_value
```

2. Multiple testing correction

2.1 Benjamini-Hochberg (FDR)

Procedure:

Sort p-values in ascending order: $p_1 \leq p_2 \leq \ldots \leq p_m$

Find largest i such that: $p_i \leq (i/m) \times \alpha$

Reject null hypotheses for $H_1, H_2, \ldots, H_i$

Implementation:

```python
from statsmodels.stats.multitest import multipletests

def benjamini_hochberg_correction(pvalues, alpha=0.05):
    """
    Apply Benjamini-Hochberg FDR correction

    Parameters:
    pvalues: array of p-values
    alpha: significance threshold

    Returns:
    reject: boolean array (True if null hypothesis rejected)
    pvals_corrected: FDR-adjusted p-values
    """
    reject, pvals_corrected, alpha_sidak, alpha_bonf = multipletests(
        pvalues,
        alpha=alpha,
        method='fdr_bh',
        is_sorted=False,
```

```
        returnsorted=False
    )

    return reject, pvals_corrected
```

Properties: - Controls False Discovery Rate (FDR) - Less conservative than Bonferroni - Recommended for exploratory analyses - Default method in NeXus

2.2 Bonferroni correction

Formula:

$$p_{corrected} = \min(p \times m, 1.0)$$

$

Where: - p = original p-value - m = number of tests

Implementation:

```
def bonferroni_correction(pvalues, alpha=0.05):
    """
    Apply Bonferroni correction

    Parameters:
    pvalues: array of p-values
    alpha: significance threshold

    Returns:
    reject: boolean array
    pvals_corrected: Bonferroni-adjusted p-values
    """
    m = len(pvalues)
    pvals_corrected = np.minimum(pvalues <i> m, 1.0)
    reject = pvals_corrected < alpha

    return reject, pvals_corrected
```

Properties: - Controls Family-Wise Error Rate (FWER) - Most conservative correction - Used for validation/confirmation - Available as optional in NeXus

2.3 Comparison of correction methods

Decision guide:

Method	Controls	Use When	Conservative	Power
Benjamini-Hochberg	FDR	Exploratory, many tests	Moderate	Higher

Method	Controls	Use When	Conservative	Power
Bonferroni	FWER	Confirmatory, few tests	High	Lower

Practical implementation in NeXus:

```yaml
multiple_testing:
  primary_method: 'fdr_bh'      # For main results
  secondary_method: 'bonferroni'  # For validation
  alpha: 0.05
  report_both: true            # Compare both methods
```

3. Network randomization

3.1 Configuration model

Purpose: Generate random networks preserving degree distribution

Algorithm: 1. Extract degree sequence from observed network 2. Create stubs (half-edges) for each node 3. Randomly connect stubs avoiding self-loops and multi-edges 4. Repeat until valid network generated

Implementation:

```python
import networkx as nx

def generate_random_network(G, seed=None):
    """
    Generate random network using configuration model

    Parameters:
    G: observed network
    seed: random seed for reproducibility

    Returns:
    G_random: random network with same degree distribution
    """
    degree_sequence = [d for n, d in G.degree()]

    # Generate random graph
    G_random = nx.configuration_model(degree_sequence, seed=seed)

    # Convert to simple graph (remove self-loops, multi-edges)
    G_random = nx.Graph(G_random)
    G_random.remove_edges_from(nx.selfloop_edges(G_random))

    return G_random
```

3.2 Null distribution generation

Procedure:

```
def generate_null_distribution(G, n_iterations=1000, metrics=['clustering', 'modularity']):
    """
    Generate null distribution for network metrics

    Parameters:
    G: observed network
    n_iterations: number of random networks to generate
    metrics: list of metrics to compute

    Returns:
    null_distributions: dict of metric -> array of values
    """
    import community as community_louvain

    null_distributions = {metric: [] for metric in metrics}

    for i in range(n_iterations):
        # Generate random network
        G_random = generate_random_network(G, seed=i)

        # Compute metrics
        if 'clustering' in metrics:
            null_distributions['clustering'].append(
                nx.average_clustering(G_random)
            )

        if 'modularity' in metrics:
            partition = community_louvain.best_partition(G_random)
            modularity = community_louvain.modularity(partition, G_random)
            null_distributions['modularity'].append(modularity)

        if 'path_length' in metrics:
            if nx.is_connected(G_random):
                null_distributions['path_length'].append(
                    nx.average_shortest_path_length(G_random)
                )

    return null_distributions
```

3.3 Convergence analysis

Monitoring convergence:

```
def check_convergence(values, window=100):
    """
```

```python
    Check if null distribution has converged

    Parameters:
    values: list of metric values from randomizations
    window: window size for computing coefficient of variation

    Returns:
    is_converged: boolean
    cv: coefficient of variation
    """
    if len(values) < window:
        return False, None

    # Compute CV over last window values
    recent_values = values[-window:]
    cv = np.std(recent_values) / np.mean(recent_values)

    # Converged if CV < 0.02 (2%)
    is_converged = cv < 0.02

    return is_converged, cv
```

Monitoring during analysis:

```python
def adaptive_randomization(G, min_iterations=500, max_iterations=2000):
    """
    Perform randomization with convergence checking

    Parameters:
    G: observed network
    min_iterations: minimum randomizations before checking convergence
    max_iterations: maximum randomizations to perform

    Returns:
    null_distributions: converged null distributions
    n_iterations: actual number of iterations performed
    """
    null_distributions = {'clustering': [], 'modularity': []}

    for i in range(max_iterations):
        G_random = generate_random_network(G, seed=i)

        # Compute metrics
        clustering = nx.average_clustering(G_random)
        null_distributions['clustering'].append(clustering)

        partition = community_louvain.best_partition(G_random)
```

```
        modularity = community_louvain.modularity(partition, G_random)
        null_distributions['modularity'].append(modularity)

        # Check convergence after minimum iterations
        if i >= min_iterations:
            converged_clustering, cv_clustering = check_convergence(
                null_distributions['clustering']
            )
            converged_modularity, cv_modularity = check_convergence(
                null_distributions['modularity']
            )

            if converged_clustering and converged_modularity:
                print(f"Converged after {i+1} iterations")
                print(f"CV clustering: {cv_clustering:.4f}")
                print(f"CV modularity: {cv_modularity:.4f}")
                break

    return null_distributions, i+1
```

3.4 P-value calculation from null distribution

Two-tailed test:

```
def calculate_pvalue_from_null(observed_value, null_distribution, alternative='two-sided'):
    """
    Calculate p-value from null distribution

    Parameters:
    observed_value: metric value from observed network
    null_distribution: array of values from random networks
    alternative: 'two-sided', 'greater', or 'less'

    Returns:
    p_value: statistical significance
    """
    n = len(null_distribution)

    if alternative == 'greater':
        # P(random >= observed)
        p_value = (np.array(null_distribution) >= observed_value).sum() / n

    elif alternative == 'less':
        # P(random <= observed)
        p_value = (np.array(null_distribution) <= observed_value).sum() / n
```

```python
    else:  # two-sided
        # P(|random| >= |observed|)
        abs_observed = abs(observed_value)
        abs_null = np.abs(null_distribution)
        p_value = (abs_null >= abs_observed).sum() / n

    # Avoid p = 0 (use 1/n as minimum)
    p_value = max(p_value, 1/n)

    return p_value
```

4. Effect size quantification

4.1 Fold Enrichment

Formula:

$$FE = \frac{(k/n)}{(K/N)} = \frac{k \cdot N}{n \cdot K}$$

Where: - k = observed overlap - n = query size - K = gene set size - N = background size

Interpretation: - FE = 1: No enrichment (as expected by chance) - FE > 1: Positive enrichment - FE < 1: Depletion

Implementation:

```python
def calculate_fold_enrichment(k, n, K, N):
    """
    Calculate fold enrichment

    Parameters:
    k: overlap size
    n: query size
    K: gene set size
    N: background size

    Returns:
    fold_enrichment: ratio of observed to expected overlap
    """
    expected = (n </i> K) / N
    fold_enrichment = k / expected if expected > 0 else 0

    return fold_enrichment
```

4.2 Gene ratio

Formula:

$$ $GR = \frac{k}{K} $$

$

Interpretation: - Proportion of gene set genes present in query - Independent of background size - Useful for comparing across datasets

4.3 Normalized Enrichment Score (GSEA)

Formula:

$$ $NES = \frac{ES}{mean(|ES_{null}|)} $$

$

Interpretation: - NES > 0: Enriched at top of ranked list - NES < 0: Enriched at bottom - |NES| > 1.0: Potentially significant

5. Validation against external datasets

5.1 Network validation

Comparison with established databases:

```
def validate_network_edges(G_observed, G_reference, node_type='gene'):
    """
    Validate network edges against reference database

    Parameters:
    G_observed: network from NeXus analysis
    G_reference: network from reference database (e.g., STRING)
    node_type: type of nodes to validate

    Returns:
    validation_metrics: dict with precision, recall, F1
    """
    # Extract edges of specified type
    observed_edges = {
        (u, v) for u, v in G_observed.edges()
        if G_observed.nodes[u]['node_type'] == node_type
        and G_observed.nodes[v]['node_type'] == node_type
    }

    reference_edges = {
        (u, v) for u, v in G_reference.edges()
        if u in G_observed and v in G_observed
    }

    # Calculate overlap
    true_positives = len(observed_edges & reference_edges)
    false_positives = len(observed_edges - reference_edges)
```

```
    false_negatives = len(reference_edges - observed_edges)

    # Metrics
    precision = true_positives / (true_positives + false_positives) if (true_positives + false_positives) > 0 else 0
    recall = true_positives / (true_positives + false_negatives) if (true_positives + false_negatives) > 0 else 0
    f1_score = 2 <i> (precision </i> recall) / (precision + recall) if (precision + recall) > 0 else 0

    return {
        'precision': precision,
        'recall': recall,
        'f1_score': f1_score,
        'true_positives': true_positives,
        'false_positives': false_positives,
        'false_negatives': false_negatives
    }
```

5.2 Enrichment validation

Comparison with published results:

```
def validate_enrichment_pathways(nexus_pathways, published_pathways, threshold=0.05):
    """
    Validate enrichment results against published findings

    Parameters:
    nexus_pathways: set of significant pathways from NeXus
    published_pathways: set of pathways from literature
    threshold: FDR threshold for significance

    Returns:
    concordance_metrics: dict with overlap statistics
    """
    overlap = nexus_pathways & published_pathways
    nexus_only = nexus_pathways - published_pathways
    published_only = published_pathways - nexus_pathways

    concordance = len(overlap) / len(nexus_pathways | published_pathways) if (nexus_pathways | published_pathways) else 0

    return {
        'concordance': concordance,
        'overlap_count': len(overlap),
        'nexus_only_count': len(nexus_only),
        'published_only_count': len(published_only),
        'overlap_pathways': list(overlap),
```

```python
        'nexus_only_pathways': list(nexus_only),
        'published_only_pathways': list(published_only)
    }
```

5.3 Cross-dataset validation

Performance across different datasets:

```python
def cross_dataset_validation(datasets, nexus_function):
    """
    Validate NeXus performance across multiple datasets

    Parameters:
    datasets: list of (data, ground_truth) tuples
    nexus_function: function to run NeXus analysis

    Returns:
    performance_metrics: dict with metrics per dataset
    """
    results = {}

    for dataset_name, (data, ground_truth) in datasets.items():
        # Run NeXus analysis
        nexus_results = nexus_function(data)

        # Compare with ground truth
        validation = validate_enrichment_pathways(
            set(nexus_results['significant_pathways']),
            set(ground_truth['pathways'])
        )

        results[dataset_name] = validation

    # Aggregate statistics
    avg_concordance = np.mean([r['concordance'] for r in results.values()])

    return {
        'per_dataset': results,
        'average_concordance': avg_concordance
    }
```

6. Performance benchmarking

6.1 Execution time profiling

Time complexity analysis:

```python
import time

def benchmark_analysis(gene_counts=[100, 500, 1000, 5000, 10000]):
    """
    Benchmark execution time vs dataset size

    Parameters:
    gene_counts: list of gene set sizes to test

    Returns:
    timing_results: dict with size -> time mapping
    """
    timing_results = {}

    for n_genes in gene_counts:
        # Generate test data
        test_genes = [f"GENE{i}" for i in range(n_genes)]

        # Measure time
        start_time = time.time()
        results = run_nexus_analysis(test_genes)
        end_time = time.time()

        timing_results[n_genes] = end_time - start_time

    return timing_results
```

Time complexity verification:

```python
from scipy.stats import linregress

def verify_linear_complexity(timing_results):
    """
    Verify linear time complexity O(n)

    Parameters:
    timing_results: dict of size -> time

    Returns:
    is_linear: boolean, R² value
    """
    sizes = np.array(list(timing_results.keys()))
    times = np.array(list(timing_results.values()))

    # Linear regression
    slope, intercept, r_value, p_value, std_err = linregress(sizes, times)
```

```
    # Check R² > 0.95 for linear relationship
    is_linear = r_value<b>2 > 0.95

    return is_linear, r_value</b>2, slope
```

6.2 Memory usage profiling

Peak memory measurement:

```
import tracemalloc

def measure_memory_usage(analysis_function, <i>args):
    """
    Measure peak memory usage during analysis

    Parameters:
    analysis_function: function to profile
    </i>args: arguments to pass to function

    Returns:
    peak_memory_mb: peak memory in megabytes
    """
    tracemalloc.start()

    # Run analysis
    result = analysis_function(*args)

    current, peak = tracemalloc.get_traced_memory()
    tracemalloc.stop()

    peak_memory_mb = peak / 1024 / 1024  # Convert to MB

    return peak_memory_mb, result
```

6.3 Scalability testing

Test with increasing data sizes:

```
def scalability_test(max_genes=10000, step=1000):
    """
    Test scalability across range of dataset sizes

    Parameters:
    max_genes: maximum gene count to test
    step: increment size
```

```
    Returns:
    scalability_report: dict with performance metrics
    """
    results = {
        'sizes': [],
        'execution_times': [],
        'memory_usage': [],
        'success': []
    }

    for n_genes in range(100, max_genes + 1, step):
        try:
            test_data = generate_test_dataset(n_genes)

            # Measure execution time
            start_time = time.time()
            analysis_result = run_nexus_analysis(test_data)
            execution_time = time.time() - start_time

            # Measure memory
            memory_mb = get_current_memory_mb()

            results['sizes'].append(n_genes)
            results['execution_times'].append(execution_time)
            results['memory_usage'].append(memory_mb)
            results['success'].append(True)

        except Exception as e:
            results['sizes'].append(n_genes)
            results['execution_times'].append(None)
            results['memory_usage'].append(None)
            results['success'].append(False)
            print(f"Failed at {n_genes} genes: {e}")

    return results
```

7. Reproducibility measures

7.1 Random seed management

All random operations seeded:

```
import random
import numpy as np

SEED = 42
```

```python
def set_random_seeds(seed=SEED):
    """
    Set all random seeds for reproducibility

    Parameters:
    seed: integer seed value
    """
    random.seed(seed)
    np.random.seed(seed)

    # For libraries that use random number generation
    try:
        import tensorflow as tf
        tf.random.set_seed(seed)
    except ImportError:
        pass

    try:
        import torch
        torch.manual_seed(seed)
    except ImportError:
        pass
```

7.2 Version control

Track software versions:

```python
import pkg_resources

def get_software_versions():
    """
    Get versions of all dependencies

    Returns:
    versions: dict of package -> version
    """
    packages = [
        'networkx', 'pandas', 'numpy', 'matplotlib',
        'seaborn', 'scipy', 'gseapy', 'statsmodels'
    ]

    versions = {}
    for package in packages:
        try:
            version = pkg_resources.get_distribution(package).version
            versions[package] = version
```

```python
    except pkg_resources.DistributionNotFound:
        versions[package] = "not installed"

return versions
```
