# Supplementary material for: NeXus: An Automated Platform for Network Pharmacology and Multi-Method Enrichment Analysis
Source: Int J Mol Sci. 2025 Nov 18;26(22):11147. doi: 10.3390/ijms262211147 (PMC12653797; doi:10.3390/ijms262211147)
Supplement: Supplementary file 1 [file ijms-26-11147-s001.zip › Supp Methods/Supplementary Methods S3-S4.pdf]

Supplementary Methods S3-S4. Network construction and topology analysis

Overview

This document provides detailed specifications for network construction algorithms and topological analysis methods implemented in NeXus v1.2. The platform constructs multi-layer biological networks representing plant-compound-gene relationships using NetworkX graph theory algorithms.

PART 1: NETWORK CONSTRUCTION (S3)

1. Graph Architecture

1.1 Graph Type Selection

Implementation: Undirected weighted graph

```
import networkx as nx
G = nx.Graph()  # Undirected graph
```

Rationale: - Biological interactions generally bidirectional - Compound-gene: compound affects gene, gene expression may influence compound activity - Plant-compound: relationship is associative, not directional - Weighted edges capture interaction strength/confidence

1.2 Node Types

Three distinct node types in multi-layer network:

Node Type	Prefix	Color	Size	Shape
Gene	gene_	Blue (#2E86AB)	500	Circle
Compound	compound_	Green (#06A77D)	700	Square
Plant	plant_	Pink (#D62246)	900	Diamond

Node attribute structure:

```
node_attributes = {
    'node_id': str,
    'node_type': str,  # 'gene', 'compound', or 'plant'
    'label': str,  # Display name
    'color': str,  # Hex color code
    'size': int,  # Visualization size
    'degree': int,  # Computed later
    'source_data': dict  # Original data reference
}
```

2. Network Layer Construction

2.1 Gene Layer

Node Creation:

```python
for gene in unique_genes:
   G.add_node(
      f"gene_{gene}",
      node_type='gene',
      label=gene,
      color='#2E86AB',
      size=500,
      biological_type='protein_coding'  # inferred or from annotation
   )
```

Gene node properties: - Identifier: HGNC symbol or database ID - Attributes: Gene name, chromosome location (if available) - Biological context: Protein-coding, ncRNA, etc.

2.2 Compound Layer

Node Creation:

```python
for compound in unique_compounds:
   G.add_node(
      f"compound_{compound}",
      node_type='compound',
      label=compound,
      color='#06A77D',
      size=700,
      chemical_type='small_molecule'
   )
```

Compound node properties: - Identifier: Common name or database ID (ChEMBL, PubChem) - Attributes: Molecular formula, weight (if available) - Source: Plant origin tracking

2.3 Plant Layer

Node Creation:

```python
for plant in unique_plants:
   G.add_node(
      f"plant_{plant}",
      node_type='plant',
      label=plant,
      color='#D62246',
      size=900,
      taxonomy='botanical_name'
   )
```

Plant node properties: - Identifier: Botanical name or common name - Attributes: Taxonomy, traditional use - Contribution: Number of associated compounds

3. Edge Construction

3.1 Compound-Gene Edges

Edge creation logic:

```
for idx, row in df.iterrows():
    if row['gene'] and row['compound']:
        gene_node = f"gene_{row['gene']}"
        compound_node = f"compound_{row['compound']}"

        if G.has_edge(gene_node, compound_node):
            # Edge exists, increment weight
            G[gene_node][compound_node]['weight'] += 1
            G[gene_node][compound_node]['sources'].append(row['source'])
        else:
            # Create new edge
            G.add_edge(
                gene_node,
                compound_node,
                weight=1.0,
                edge_type='compound_gene',
                interaction='targets',
                sources=[row['source']],
                confidence=1.0  # default or from data
            )
```

Edge attributes: - weight: Interaction strength (1.0 default, can be literature-derived) - edge_type: 'compound_gene' - interaction: 'targets', 'regulates', 'binds' - confidence: Reliability score (0-1) - sources: Data sources or references

3.2 Plant-Compound Edges

Edge creation logic:

```
for idx, row in df.iterrows():
    if row['plant'] and row['compound']:
        plant_node = f"plant_{row['plant']}"
        compound_node = f"compound_{row['compound']}"

        if not G.has_edge(plant_node, compound_node):
            G.add_edge(
                plant_node,
                compound_node,
                weight=1.0,
                edge_type='plant_compound',
                relationship='contains',
```

```
        concentration='unknown'  # can be added from data
    )
```

Edge attributes: - weight: 1.0 (presence/absence) - edge_type: 'plant_compound' - relationship: 'contains', 'produces' - concentration: Quantitative data if available

3.3 Multi-Edge Handling

Policy: Multiple evidence for same interaction increases weight

```
# If gene-compound pair appears multiple times
weight = number_of_occurrences
confidence = min(1.0, base_confidence * sqrt(occurrences))
```

4. Network Integrity Checks

4.1 Connectivity Validation

Check 1: Orphan node detection

```
orphan_nodes = [node for node in G.nodes() if G.degree(node) == 0]
```

Action: Log warning if >5% nodes are orphans

Check 2: Component analysis

```
components = list(nx.connected_components(G))
largest_component = max(components, key=len)
```

Action: Log if >1 component, report size of largest

Check 3: Bipartite validation

```
# Verify gene-compound layer is bipartite
gene_nodes = {n for n in G.nodes() if G.nodes[n]['node_type'] == 'gene'}
compound_nodes = {n for n in G.nodes() if G.nodes[n]['node_type'] == 'compound'}
is_bipartite = nx.is_bipartite_node_set(G, gene_nodes)
```

4.2 Relationship Validation

Maximum degree check:

```
max_degree = max(dict(G.degree()).values())
if max_degree > 1000:
    # Warning: Possible hub node or data error
    log_warning(f"Node with degree {max_degree} detected")
```

Minimum subgraph size:

```
if G.number_of_nodes() < 10:
    log_warning("Network very small, results may not be reliable")
```

PART 2: TOPOLOGY ANALYSIS (S4)

5. Centrality Metrics

5.1 Degree Centrality

Definition: Number of direct connections a node has

Computation:

```
degree_centrality = nx.degree_centrality(G)
```

Formula:

$$ $C_D(v) = \frac{deg(v)}{n - 1} $$

$

Where: - deg(v) = number of edges connected to node v - n = total number of nodes

Interpretation: - High degree = hub node, highly connected - Critical for identifying key compounds/genes

5.2 Betweenness Centrality

Definition: Frequency of node appearing on shortest paths

Computation:

```
betweenness_centrality = nx.betweenness_centrality(G, weight='weight')
```

Formula:

$$ $C_B(v) = \sum_{s \neq v \neq t} \frac{\sigma_{st}(v)}{\sigma_{st}} $$

$

Where: - σ_st = total shortest paths from s to t - σ_st(v) = shortest paths passing through v

Interpretation: - High betweenness = bridging node, controls information flow - Important for identifying regulatory compounds

Performance optimization:

```
# For large networks (>10,000 nodes), use approximation
if G.number_of_nodes() > 10000:
    betweenness = nx.betweenness_centrality(G, k=1000)  # Sample 1000 nodes
```

5.3 Closeness Centrality

Definition: Average distance to all other nodes

Computation:

```
closeness_centrality = nx.closeness_centrality(G, distance='weight')
```

Formula:

$$ $C_C(v) = \frac{n - 1}{\sum_{u \neq v} d(v, u)} $$

$

Where: - d(v, u) = shortest path distance between v and u - n = number of nodes

Interpretation: - High closeness = central position, quick access to network - Indicates global importance

5.4 Eigenvector Centrality

Definition: Influence based on connections to high-degree nodes

Computation:

```
try:
    eigenvector_centrality = nx.eigenvector_centrality(G, max_iter=1000, weight='weight')
except nx.PowerIterationFailedConvergence:
    # Fallback to approximation
    eigenvector_centrality = nx.eigenvector_centrality_numpy(G, weight='weight')
```

Formula:

$$ $x_v = \frac{1}{\lambda} \sum_{u \in N(v)} x_u $$

$

Where: - λ = largest eigenvalue - N(v) = neighbors of v - x_u = centrality of neighbor u

Interpretation: - High eigenvector = connected to important nodes - Captures indirect influence

6. Network Topology Metrics

6.1 Clustering Coefficient

Local clustering coefficient:

```
clustering_coefficients = nx.clustering(G, weight='weight')
average_clustering = nx.average_clustering(G, weight='weight')
```

Formula:

$$ $C_i = \frac{2e_i}{k_i(k_i - 1)} $$

$

Where: - e_i = number of edges between neighbors of node i - k_i = degree of node i

Weighted version:

$$ $C_i^w = \frac{1}{s_i(k_i - 1)} \sum_{j,k} \frac{w_{ij} + w_{ik}}{2} a_{ij} a_{ik} a_{jk} $$

$

Interpretation: - High clustering = dense local neighborhoods - Indicates modular structure

6.2 Path Length Analysis

Average shortest path length:

```python
if nx.is_connected(G):
    avg_path_length = nx.average_shortest_path_length(G, weight='weight')
else:
    # For disconnected graphs, use largest component
    largest_cc = max(nx.connected_components(G), key=len)
    subG = G.subgraph(largest_cc)
    avg_path_length = nx.average_shortest_path_length(subG, weight='weight')
```

Formula:

$$ $L = \frac{1}{n(n-1)} \sum_{i \neq j} d(i, j) $$

$

Interpretation: - Low path length = small-world property - Efficient signal propagation in network

6.3 Degree Distribution

Computation:

```python
degree_sequence = [d for n, d in G.degree()]
degree_count = Counter(degree_sequence)
```

Power-law fitting:

```python
from scipy.stats import powerlaw
import numpy as np

degrees = np.array(degree_sequence)
# Fit power law: P(k) ~ k^(-γ)
fit_alpha, fit_loc, fit_scale = powerlaw.fit(degrees[degrees > 0])
```

Scale-free test: - Calculate R^2 of power-law fit - $R^2 > 0.8$ suggests scale-free topology - Characteristic of biological networks

7. Community Detection

7.1 Louvain Algorithm

Implementation:

```python
import community as community_louvain

# Convert to format for python-louvain
partition = community_louvain.best_partition(G, weight='weight', resolution=1.0)

# Calculate modularity
modularity = community_louvain.modularity(partition, G, weight='weight')
```

Modularity formula:

$$ $Q = \frac{1}{2m} \sum_{ij} \left[A_{ij} - \frac{k_i k_j}{2m} \right] \delta(c_i, c_j) $$

$

Where: - m = total number of edges - A_ij = adjacency matrix - k_i = degree of node i - δ(c_i, c_j) = 1 if nodes i, j in same community, 0 otherwise

Resolution parameter optimization:

```
# Test multiple resolution values
resolutions = [0.5, 0.8, 1.0, 1.2, 1.5]
best_modularity = -1
best_partition = None

for res in resolutions:
    partition = community_louvain.best_partition(G, weight='weight', resolution=res)
    mod = community_louvain.modularity(partition, G, weight='weight')

    if mod > best_modularity:
        best_modularity = mod
        best_partition = partition
```

7.2 Module Characterization

Functional analysis per module:

```
for community_id in set(partition.values()):
    # Get nodes in this community
    community_nodes = [n for n, c in partition.items() if c == community_id]

    # Extract genes in this community
    genes_in_community = [
        G.nodes[n]['label']
        for n in community_nodes
        if G.nodes[n]['node_type'] == 'gene'
    ]

    # Perform enrichment analysis on genes_in_community
    module_enrichment = perform_enrichment(genes_in_community)
```

8. Statistical Significance Testing

8.1 Random Network Generation

Configuration model:

```
def generate_random_network(G, seed=None):
    """
    Generate random network preserving degree distribution
```

```python
    """
    degree_sequence = [d for n, d in G.degree()]
    random_G = nx.configuration_model(degree_sequence, seed=seed)

    # Remove self-loops and multi-edges
    random_G = nx.Graph(random_G)
    random_G.remove_edges_from(nx.selfloop_edges(random_G))

    return random_G
```

Number of randomizations: 1,000 (default, configurable)

8.2 Null Distribution Comparison

Process:

```python
# Observed network metrics
observed_clustering = nx.average_clustering(G)
observed_path_length = nx.average_shortest_path_length(G)
observed_modularity = best_modularity

# Generate null distribution
random_clustering = []
random_path_length = []
random_modularity = []

for i in range(1000):
    random_G = generate_random_network(G, seed=i)

    random_clustering.append(nx.average_clustering(random_G))

    if nx.is_connected(random_G):
        random_path_length.append(nx.average_shortest_path_length(random_G))

    rand_partition = community_louvain.best_partition(random_G)
    random_modularity.append(community_louvain.modularity(rand_partition, random_G))

# Calculate p-values
p_clustering = (np.array(random_clustering) >= observed_clustering).sum() / 1000
p_modularity = (np.array(random_modularity) >= observed_modularity).sum() / 1000
```

Significance threshold: $p < 0.05$

9. Network Export Formats

9.1 GEXF Format

Graph Exchange XML Format - For Gephi visualization

```
nx.write_gexf(G, "network.gexf")
```

Advantages: - Preserves all node/edge attributes - Compatible with Gephi, Cytoscape - Human-readable XML

9.2 JSON Format

JavaScript Object Notation - For web applications

```
from networkx.readwrite import json_graph
network_json = json_graph.node_link_data(G)

with open('network.json', 'w') as f:
    json.dump(network_json, f, indent=2)
```

9.3 Adjacency Matrix

For mathematical analysis

```
adjacency_matrix = nx.adjacency_matrix(G, weight='weight')
```

10. Performance Benchmarks

10.1 Construction Time

Time complexity: $O(n + m)$ - n = number of nodes - m = number of edges

Empirical times: - 100 nodes, 500 edges: ~0.5 seconds - 1,000 nodes, 5,000 edges: ~1.2 seconds - 10,000 nodes, 50,000 edges: ~8 seconds

10.2 Memory Usage

Space complexity: $O(n + m)$

Empirical memory: - 100 nodes: ~50 KB - 1,000 nodes: ~500 KB - 10,000 nodes: ~5 MB

10.3 Centrality Computation Time

Metric	Time Complexity	1K nodes	10K nodes
Degree	$O(n)$	<0.1s	<0.5s
Betweenness	$O(n^2)$	~2s	~80s
Closeness	$O(n^2)$	~1.5s	~60s
Eigenvector	$O(n^2)$	~0.5s	~10s
