# Supplementary material for: NeXus: An Automated Platform for Network Pharmacology and Multi-Method Enrichment Analysis
Source: Int J Mol Sci. 2025 Nov 18;26(22):11147. doi: 10.3390/ijms262211147 (PMC12653797; doi:10.3390/ijms262211147)
Supplement: Supplementary file 1 [file ijms-26-11147-s001.zip › Supp Tables/Supplementary Table S1.pdf]

Supplementary Table S1. Detailed characterization of high-connectivity compounds identified in network analysis, representing 15.3% of total compounds with potential roles as hub compounds or multi-target agents.

Compound ID	Compound name	Degree (connectivity)	Source plant(s)	Target genes (selected)	Top enriched pathways	P-value
C001	Quercetin	23	Plant 1, Plant 2	MAPK1, MAPK3, AKT1, PIK3CA, EGFR, VEGFA, IL6, TNF, NOS3, PTGS2, TP53, CASP3, BCL2, NFKB1, JUN, FOS, ESR1, AR, PPARG, RELA (+ 3 more)	MAPK signaling pathway	1.3×10^{-9}
					PI3K-Akt signaling pathway	2.7×10^{-8}
					TNF signaling pathway	4.2×10^{-7}
C007	Kaempferol	18	Plant 1, Plant 3	MAPK1, APK14, AKT1, EGFR, IL6, TNF, PTGS2, TP53, CASP3, BCL2, NFKB1, JUN, ESR1, PPARG, RELA, STAT3, MMP9, HIF1A	Inflammatory mediator regulation	3.8×10^{-8}
					Apoptosis pathway	1.2×10^{-7}
					Cancer pathway	5.6×10^{-7}
C012	Luteolin	17	Plant 2	MAPK1, AKT1, PIK3CA, IL6, TNF, PTGS2, TP53, CASP3, NFKB1, JUN, ESR1, PPARG, STAT3, MMP9, HIF1A, VEGFA, NOS2	NF-kappa B signaling pathway	2.1×10^{-8}
					PI3K-Akt signaling pathway	6.7×10^{-8}
					HIF-1 signaling pathway	1.4×10^{-6}

Compound ID	Compound name	Degree (connectivity)	Source plant(s)	Target genes (selected)	Top enriched pathways	P-value
C015	Beta-sitosterol	15	Plant 1, Plant 2, Plant 3	PTGS2, TNF, IL6, NFKB1, RELA, IL1B, PPARG, NOS2, ICAM1, VCAM1, CCL2, IL10, STAT3, TLR4, HMGCR	Inflammatory response	5.3×10^{-9}
					Lipid metabolism	2.8×10^{-7}
					NF-kappa B signaling	7.1×10^{-7}
C023	Stigmasterol	12	Plant 2, Plant 3	PTGS2, TNF, IL6, PPARG, NOS2, ICAM1, IL1B, CCL2, TLR4, SREBF1, HMGCR, INSIG1	Steroid biosynthesis	8.4×10^{-8}
					PPAR signaling	1.6×10^{-7}
					Toll-like receptor signaling	4.2×10^{-6}
