# Supplementary material for: NeXus: An Automated Platform for Network Pharmacology and Multi-Method Enrichment Analysis
Source: Int J Mol Sci. 2025 Nov 18;26(22):11147. doi: 10.3390/ijms262211147 (PMC12653797; doi:10.3390/ijms262211147)
Supplement: Supplementary file 1 [file ijms-26-11147-s001.zip › Supp Tables/Supplementary Table S2.pdf]

Supplementary Table S2. Complete list of NeXus v1.2 Python modules with descriptions and functions.

Module name	Primary function	Key classes/ methods	Dependencies
run_nexus.py	Master analysis script with command-line interface	NEXUSMasterPipeline class, main() function	All modules below
network_builder.py	Multi-layer network construction	NetworkBuilder, build_network(), calculate_centrality()	NetworkX, pandas
enrichment_comprehensive.py	ORA, GSEA, GSVA implementation	EnrichmentAnalyzer, run_gsea(),run_gsva()	gseapy, scipy
publication_figures.py	300 DPI figure generation	PublicationFigureGenerator, create_figure_X()	matplotlib, seaborn
data_validator.py	Input data validation and quality control	DataValidator, validate_file(), validate_genes()	pandas, re
input_parser.py	CSV parsing and format detection	InputParser, parse_csv(), detect_format()	pandas
gene_selector.py	Gene selection and documentation	GeneSelector, select_genes(), generate_report()	pandas
gsea_analyzer.py	GSEA-specific implementation	GSEAAnalyzer, run_enrichment()	gseapy
enrichment_visualizer.py	Enrichment plot generation	EnrichmentVisualizer, create_barplot(), create_heatmap()	matplotlib, seaborn
network_visualizer.py	Network diagram generation	NetworkVisualizer, create_network_plot()	NetworkX, matplotlib
network_enrichment_integration.py	Integration layer for network and enrichment	integrate_network_enrichment()	NetworkX, pandas
improved_output_manager.py	File organization and report generation	ImprovedOutputManager, save_results()	pathlib, json

Module name	Primary function	Key classes/ methods	Dependencies
method_selector.py	Automatic method recommendation	MethodSelector, recommend_method()	pandas
analysis_types.py	Analysis type detection	detect_analysis_type()	pandas
simple_pipeline.py	Simplified workflow script	run_simple_analysis()	Selected modules
complete_pipeline.py	Advanced pipeline (development)	CompletePipeline class	All modules
